# Genetic Differences between the Determinants of Lipid Profile Phenotypes in African and European Americans: The Jackson Heart Study

**DOI:** 10.1371/journal.pgen.1000342

**Published:** 2009-01-16

**Authors:** Rahul C. Deo, David Reich, Arti Tandon, Ermeg Akylbekova, Nick Patterson, Alicja Waliszewska, Sekar Kathiresan, Daniel Sarpong, Herman A. Taylor, James G. Wilson

**Affiliations:** 1 Department of Genetics, Harvard Medical School, Boston, Massachusetts, United States of America; 2 Cardiology Division, Massachusetts General Hospital, Harvard Medical School, Boston, Massachusetts, United States of America; 3 Department of Medicine, Harvard Medical School, Boston, Massachusetts, United States of America; 4 Program in Medical and Population Genetics, Broad Institute of Harvard and MIT, Cambridge, Massachusetts, United States of America; 5 Department of Medicine, University of Mississippi Medical Center, Jackson, Mississippi, United States of America; 6 Jackson State University, Jackson, Mississippi, United States of America; 7 Tougaloo College, Tougaloo, Mississippi, United States of America; 8 G. V. (Sonny) Montgomery Veterans Affairs Medical Center, Jackson, Mississippi, United States of America; Queensland Institute of Medical Research, Australia

## Abstract

Genome-wide association analysis in populations of European descent has recently found more than a hundred genetic variants affecting risk for common disease. An open question, however, is how relevant the variants discovered in Europeans are to other populations. To address this problem for cardiovascular phenotypes, we studied a cohort of 4,464 African Americans from the Jackson Heart Study (JHS), in whom we genotyped both a panel of 12 recently discovered genetic variants known to predict lipid profile levels in Europeans and a panel of up to 1,447 ancestry informative markers allowing us to determine the African ancestry proportion of each individual at each position in the genome. Focusing on lipid profiles—HDL-cholesterol (HDL-C), LDL-cholesterol (LDL-C), and triglycerides (TG)—we identified the lipoprotein lipase (*LPL*) locus as harboring variants that account for interethnic variation in HDL-C and TG. In particular, we identified a novel common variant within *LPL* that is strongly associated with TG (*p* = 2.7×10^−6^) and explains nearly 1% of the variability in this phenotype, the most of any variant in African Americans to date. Strikingly, the extensively studied “gain-of-function” *S447X* mutation at *LPL*, which has been hypothesized to be the major determinant of the *LPL-*TG genetic association and is in trials for human gene therapy, has a significantly diminished strength of biological effect when it is found on a background of African rather than European ancestry. These results suggest that there are other, yet undiscovered variants at the locus that are truly causal (and are in linkage disequilibrium with *S447X*) or that work synergistically with *S447X* to modulate TG levels. Finally, we find systematically lower effect sizes for the 12 risk variants discovered in European populations on the African local ancestry background in JHS, highlighting the need for caution in the use of genetic variants for risk assessment across different populations.

## Author Summary

Single-base changes in DNA can affect biochemical measures, such as blood cholesterol or lipid levels. Such changes or “variants” can be associated with a trait either because they cause the trait or because they are linked to other causal variants. In either case, the associated variant(s) may be useful in predicting the trait. The chromosomes in which DNA is packaged cross over and recombine with each other in each generation, so that in historically separate populations, such as Africans and Europeans, the patterns of genetic linkage between variants differ. In the current study, we analyzed a large group of African Americans, testing genetic variants that had been associated with cholesterol and lipid levels in European-derived populations to assess their predictive value on two different genetic backgrounds within the same cohort. The ability of some variants to predict cholesterol or lipid traits was strongly dependent on genetic background, indicating that they may be tightly linked to other causal variant(s) in European populations and may not, themselves, be directly responsible for trait variability. We conclude that the predictive value of specific variants for risk assessment can differ critically across populations.

## Introduction

A main motivation for genome-wide association studies (GWAS) of disease phenotypes has been the promise of identifying markers of disease risk, which may augment the prognostic value of conventional clinical measures [1],[2]. Although many such variants have been identified, the corresponding studies have been based almost entirely in populations of European descent. Thus, it remains to be seen if these markers will have prognostic utility in admixed individuals, such as African or Hispanic Americans, in whom each chromosome is likely to be a mosaic of blocks of DNA from different ancestral populations. African Americans, for example, carry chromosomal segments that are derived predominantly from West African and European American ancestral populations. These two populations have been subject to differing demographic history, which has led to differences in allelic frequencies and patterns of linkage disequilibrium across the genome [3],[4].

We have previously used a form of whole-genome scanning known as admixture mapping to search for genetic variants that differ markedly in frequency between continental populations and that also contribute to disease risk [5]–[9]. In persons of recently mixed ancestry, each region of the genome is mapped according to ancestral origin using a panel of markers that are highly differentiated in frequency between West Africans and European Americans. The resulting maps are then analyzed to identify genomic regions where individuals with disease have a marked deviation in the proportion of one of the parental ancestries from the genome–wide average [10]–[13]. Such regions in principle should contain disease variants.

In the current work, we have investigated to what extent the local ancestry information used in admixture mapping can inform genetic association analysis in African Americans. We chose lipid and cholesterol levels as our phenotypes of interest for several reasons: 1) they represent the major modifiable risk factors for coronary heart disease; 2) several GWAS of lipid profiles have been conducted recently in populations of European descent, yielding multiple potential risk variants [14]–[18]; and 3) the lipoprotein profiles of African Americans and European Americans differ, beginning in childhood [19] and persisting into adulthood [20], with African Americans having lower levels of triglycerides and very low density lipoprotein cholesterol, and higher levels of HDL-C and apolipoprotein A-I. We have incorporated local ancestry estimates into genetic association analysis of cholesterol and TG phenotypes in the Jackson Heart Study (JHS) [21]. We show, herein, how using such an analytic approach in admixed populations can inform an understanding of the genetic determinants of complex disease.

## Results

We studied a data set of 4605 individuals from the JHS who were consented for genotype analysis. The JHS is a long term, community-based observational study that was developed as an extension of the Jackson, MS cohort of the Atherosclerosis Risk in Communities (ARIC) study, with the support of the National Heart, Lung, and Blood Institute (NHLBI) [21]. It is designed to investigate the factors that contribute to the very high prevalence of common complex diseases among African Americans in Mississippi, including hypertension, diabetes mellitus, myocardial infarction, congestive heart failure, LVH, stroke, peripheral vascular disease, and chronic renal disease. Hypercholesterolemia is particularly prevalent in this population, with rates exceeding 35% for individuals above the age of 50 [22]. Of the 4605 individuals, 1448 are part of a family study and therefore include relatives within the study. We were able to genotype successfully 4464 individuals using one of two panels of >1400 markers selected for high differences in frequency between European Americans and West Africans [7]. Demographic characteristics are shown in Table 1.

**Table 1:** Demographic Characteristics for the Genotyped Jackson Heart Study Participants.

	All	Unrelated	Unrelated with only African local ancestry at *LPL*	Unrelated with European local ancestry at *LPL*
**Number**	4464	3300	1887	728
**Male (%)**	36.8	37.8	37.4	37.4
**Age (mean - years, ±std.)**	55±13	56±11	57±12	57±12
**BMI (mean - kg/m^2^ ±std.)**	32±7	32±7	32±7	32±7
**Type II DM (%)**	18.9	20.4	20.6	19.6
**Cholesterol or TG-Lowering Medication (%)**	12.5	13.6	13.8	13.7
**LDL-Cholesterol: unmedicated | medicated participants (mg/dL)**	127±36 | 113±33	128±37 | 113±33	126±36 | 112±35	131±37 | 119±33
**HDL-Cholesterol: unmedicated | medicated participants (mg/dL)**	51±14 | 51±14	52±15 | 52±14	52±15 | 52±15	51±15 | 49±15
**Serum Triglycerides: unmedicated | medicated participants (mg/dL)**	106±81 | 129±151	107±83 | 130±164	106±95 | 120±78	110±68 | 156±281
**Overall African Ancestry (%)**	83±9	82±9	85±7	76±7

Characteristics are shown for the 4464 genotyped participants, the 3300 unrelated individuals, the 1887 unrelated individuals with homozygous African local ancestry at the *LPL* locus, and the 728 individuals with at least one ancestral European allele at *LPL*.

### African Ancestry Is Significantly Associated with Triglyceride and HDL-Cholesterol Levels and Weakly Associated with LDL-Cholesterol Levels

Using the ANCESTRYMAP software [10], we can make precise estimates of individual ancestry in admixed individuals, which can be correlated with continuous and dichotomous phenotypes (see Methods). In the 4464 genotyped JHS participants, we found a mean African ancestry of 83±9% (Table 1). We assessed the association of overall ancestry (hereafter called global ancestry, in contrast to local ancestry of a chromosomal segment) with each of our three traits by linear regression (see Methods). Analysis was limited to unrelated individuals, with one individual selected randomly from each family, to avoid correlations arising from relatedness. One hundred separate lists of unrelated individuals were generated by repeated random sampling. We also performed logistic regression, comparing the top and bottom quintile of the lipid distributions, to see if there were differences in ancestry for extremes of the population.

After adjusting for common covariates, which accounted for 10% of the TG variance, increased African ancestry was significantly associated with decreased triglyceride levels (Table 2) in both linear (p = 6.5×10^−5^) and logistic regression analysis (p = 0.0046). For every 10% decrease in African Ancestry, there is a 3.9±0.9% increase in triglyceride level.

**Table 2:** Individual African-European Ancestry is associated with Triglyceride and Cholesterol Levels.

	p-value: Population-wide	p-value: Comparison of Upper and Lower Quintiles of Trait
**TG** 1	6.5×10^−5^	0.0046
**HDL-C** 2	0.00090	0.017
**LDL-C** 3	0.40	0.027

1 Adjusted for age, age^2^, gender, bmi, bmi^2^, type 2 diabetes (dm2), smoking.

2 Adjusted for gender, bmi, bmi^2^, dm2.

3 Adjusted for age, age^2^, gender, bmi, bmi^2^, dm2, smoking; individuals on cholesterol-lowering medication were excluded.

Linear and logistic regressions were used to estimate the strength of association of individual African/European ancestry with triglycerides and the cholesterol traits.

As shown in Table 2, HDL-C is also significantly associated with African ancestry in both linear (p = 0.0009) and logistic (p = 0.017) regression analysis (0.74 mg/dl HDL-C decrease per 10% decrease in African ancestry). For LDL-C, we included individuals who were not on cholesterol-lowering medications (n = 3521). LDL-C shows a significant association only in a comparison of upper and lower quintiles (p = 0.027). Decreased LDL-C levels were associated with increased African ancestry.

Our observed association of increased African ancestry with increased HDL-C and decreased serum triglycerides and LDL-C is in keeping with that seen in other cohorts [23],[24], although we were better able to quantify these relationships given the large size of the study population and greater informativeness of the marker panel. These findings support the hypothesis that some of the population variation in lipid traits is indeed genetic, and can be attributed to variants that have different frequencies in African and European populations. Furthermore, this lipid profile of elevated HDL-C, decreased triglycerides, and decreased LDL-C would typically be considered protective towards atherosclerosis and motivates a search for contributing genetic variants.

### Whole-Genome Admixture Mapping Does Not Identify Suggestive or Significant Loci for TG, HDL-C, or LDL-C Levels

We conducted a genome-wide admixture scan by genotyping our 4464 individuals with the markers informative for ancestry as described above. We then used the ANCESTRYMAP software to estimate the probability that each chromosomal segment in the genome arose from either a European or African ancestor, and evaluated the association of this ancestry estimate with TG/cholesterol traits. The ANCESTRYMAP software is optimized for dichotomous variables, so we defined cases and controls for each of the three traits as the top and bottom quintiles. Thus in distinction to the quantitative variable analysis shown above, we have used a categorical variable for admixture mapping. Table S1 shows the results of the whole genome scan for all admixture runs. Despite the promising association of global ancestry with TG, HDL-C and LDL-C, we find no peaks meeting either our suggestive or significant thresholds of association.

### Exclusion of 96–100% of the Genome as Contributing Substantially to Interethnic Variation in Lipid Profile Traits

To quantify the lack of association of lipid profile phenotypes with ancestry, we constructed an exclusion map to rule out loci in the genome as contributing substantially to ancestry-related risk of being in the top or bottom quintile (categorical variable) for each trait. For each position in the genome and for each trait we estimated a credible interval for the factor by which the risk for the phenotype due to African (or European) ancestry differs from that due to the opposite ancestry. For all traits analyzed, and at a significance of p<0.05, 96–100% of the genome could be excluded as bearing risks >1.5-fold due to African or European ancestry (Table S2), suggesting that no single gene makes such a strong contribution to interethnic differences in TG and cholesterol traits. We also constructed a more traditional exclusion map, excluding markers for each trait for which the LOD score was <−2 (Figure S1) for a risk model of 1.5; this also excluded 82–89% of the genome. However, our ability to exclude genetic ancestry effects in the range of 1.2–1.5-fold risk, as seen for most common disease variants identified to date [25], is much less (48–59% of genome). Thus we sought other, integrative methods to identify genetic determinants of interethnic variation.

### Evaluation of Local Ancestry at Validated Genes that Influence Lipid Levels Suggests Multiple Loci Contributing to Population Differences in Lipid Profiles

Whole-genome scanning by admixture mapping and GWAS has led to the identification of many new disease-associated gene variants. However, the stringent criteria for declaring genome-wide significance, resulting from the low prior probability of the multiple hypotheses tested (>1000 in admixture mapping; >100,000 in GWAS), have limited the number of peaks followed up, and minimal overall heritability has been explained for most complex traits. Many loci that do not meet genome-wide significance might in fact be found to be associated with the trait of interest if fine-mapping strategies were pursued. Prioritization of such follow-up can be done by integrating data beyond the genome scan itself, such as meta-analysis with other GWAS [15],[16], integration of linkage data with GWAS [26], or prioritization using literature-based searches for candidates [9].

Five recent manuscripts [14]–[18] have identified and/or validated 19 loci influencing TG, HDL-C and LDL-C levels in whole genome association analyses. We determined local ancestry at the most strongly associated SNP at each of these loci in our cohort to see whether local European or African ancestry is associated with variation in TG/cholesterol levels. A significant association would not only suggest that the loci are important for lipid variation in the JHS population, but that ethnic differences in lipid profile could be attributable to frequency differences in the associated risk alleles.

Table 3 shows p-values for the association of local ancestry with the three lipid and cholesterol traits. Of the 27 associations tested, ancestry was associated with TG, HDL-C or LDL-C at three loci with p<0.05, including one at the p<0.005 level. Although none of these associations would meet a p<0.05 significance threshold with strict Bonferroni correction (0.0018 for 27 hypotheses tested), we propose that for such strongly validated genes even a weakly positive result may warrant further investigation.

**Table 3:** Evaluation of the Association of Serum Lipid and Cholesterol Levels with Local Ancestry at 19 Lipid Loci Identified or Validated in Recent GWAS.

Trait	Gene	p-value
**LDL**	***APOB***	**0.0031**
	*APOE*	0.812
	*HMGCR*	0.858
	*LDLR*	0.843
	*MVK*	0.826
	*NCAN*	0.687
	*PCSK9*	0.365
	*SORT1*	0.475
	*TRIB1*	0.224
**HDL**	*ABCA1*	0.71
	*APOA5*	0.37
	*CETP*	0.65
	*GALNT2*	0.47
	*LIPC*	0.23
	*LIPG*	0.11
	***LPL***	**0.012**
	*MLXIPL*	0.75
	*TRIB1*	0.61
**TG**	*ANGPTL3*	0.81
	*APOA5*	0.86
	*APOB*	0.07
	*GALNT2*	0.37
	***GCKR***	**0.013**
	*LPL*	0.14
	*MLXIPL*	0.19
	*NCAN*	0.86
	*TRIB1*	0.83

Local ancestry values were estimated using ANCESTRYMAP and the strength of the association of local ancestry with each trait was evaluated by linear regression as in Table 2. Associations significant at the p<0.05 level are shown in bold.

Our local ancestry association analysis identified *LPL* (p = 0.01, HDL-C), *GCKR* (p = 0.01, TG), and *ApoB* (p = 0.003, LDL-C) as plausible genes for interethnic variation in lipid profile. Of these, *LPL* also showed suggestive association with TG (p = 0.14). Furthermore, African Americans have been shown to have higher LPL activity than European Americans, and in the same study, inclusion of LPL activity in regression models rendered the association of ethnicity with triglyceride levels non-significant [27]. We therefore decided to pursue fine mapping of *LPL* to seek specific variants that contribute to interethnic variation in TG and HDL-C levels.

### Fine-Mapping of *LPL* Identifies Frequency-Differentiated Variants for TG, HDL-C, and LDL-C

Using Tagger [28], we selected a dense panel of SNPs spanning the *LPL* locus and extending 10 kB upstream of the transcriptional start site and 15 kb downstream of rs10096633 (see below). Tagging SNPs were selected at an r^2^ of 1.0 with MAF of ≥2% in the Yoruba West African HapMap population (YRI). We then forced these SNPs into Tagger to tag this region in CEU at an r^2^ of 1.0 with MAF of ≥5%. All lipid profile GWAS to date [14]–[18] have found extremely strong association of TG with the well-known rs328 SNP or a perfect proxy. We therefore included 2 perfect proxies for this SNP in CEU, rs325 and rs17482753, yielding a total of 95 SNPs.

Both global and local ancestries can be significant confounders of the association of a SNP with a phenotype in an admixed population, such as African Americans. If a trait such as TG or HDL-C is strongly associated with overall African or European ancestry, then any SNP with frequency differentiation between Europeans and Africans may appear falsely associated with the trait, a problem known as population stratification. This confounding can be corrected by including a term for global ancestry in the regression model. Locally within the genome, however, strong admixture linkage disequilibrium (i.e. blocks of shared ancestry) can extend over many centimorgans. If the true risk variant differs in frequency between European Americans and Africans, any SNP within the block of admixture linkage disequilibrium (LD) may appear causally related to the trait if it too is sufficiently differentiated in frequency to be informative for local ancestry. This confounding occurs even after correcting for global ancestry and is in fact the basis for admixture mapping. Fortunately, it can also be addressed, by inclusion of a term for local ancestry in the regression model. A final problem is that for many genes, the pattern of LD varies depending on the ancestral origin of the chromosomal segment. In general, blocks of LD are much shorter in historically large populations such as Africans than in historically smaller populations like Europeans. Thus a SNP that is strongly associated with a trait of interest in one population may show little or no association in another population because of differing patterns of LD. This may help identify the actual risk variant since, within an admixed population, a lone causal variant would be expected to have a similar effect size on any local ancestral background.

In our cohort, we calculated a linear regression residual for each trait, where the regression model included the covariates described above as well as global ancestry for each individual. This residual was tested for association by stepwise regression with local ancestry, the SNP genotype (in an additive model), and a term for the SNP genotype-local ancestry interaction, computed as a product of the local-ancestry term and SNP genotype (see Methods). Statistical significance for genotype, and genotype-local ancestry interaction were evaluated using ANOVA, with nested regression models. As another approach to potential confounding by differing LD patterns, we tested association of each SNP separately in a subpopulation of 1860 individuals who had >95% probability of two African ancestral chromosomes in the region (JHS-AFR-2*_LPL_*), thus minimizing heterogeneity in local ancestry background, and a subpopulation of 780 individuals with >95% probability of at least one European ancestral allele (JHS-EUR-1_2*_LPL_*, see Table 1 for demographic characteristics of these subgroups). We also identified a small subgroup of 65 individuals with >95% probability of two European ancestral alleles (JHS-EUR-2*_LPL_*) for calculation of allele frequencies and linkage disequilibrium parameters (Table 4, Table S3).

**Table 4:** Effect size of *LPL* variants on TG levels.

SNP	Position	f_afr	f_eur	f_all	p_trg_all	p_int_trg	p_trg_afr	effect_all±se	effect_afr±se	effect_eur±se
**rs10096633**	19875201	0.50	0.82	0.57	**2.7E-06**	0.15	7.1E-03	6.2±1.2	4.5±1.6	6.6±3.1
**rs1011685**	19875049	0.95	0.87	0.93	**2.0E-05**	**0.027**	0.25	11.5±2.5	4.4±3.6	21.5±4.5
**rs325**	19863608	0.94	0.86	0.93	**4.5E-05**	**0.033**	0.23	10.3±2.4	4.3±3.4	19.3±4.4
**rs17482753**	19876926	0.95	0.85	0.94	**4.9E-05**	**0.023**	0.33	10.8±2.5	3.7±3.6	21.4±4.5
**rs328**	19864004	0.94	0.86	0.93	**7.1E-05**	**0.050**	0.22	10.2±2.4	4.5±3.4	18.6±4.5
**rs12679834**	19864713	0.92	0.86	0.91	**2.7E-04**	**0.013**	0.33	8.3±2.1	3.1±3.0	17.8±4.3
**rs327**	19863816	0.55	0.68	0.59	7.8E-04	0.86	8.3E-03	4.3±1.2	4.4±1.6	3.7±2.8
**rs1569209**	19874450	1.00	0.90	0.98	9.9E-04	0.86	0.62	16.6±4.7	17.3±32.5	18.9±5.6
**rs13702**	19868772	0.43	0.66	0.48	1.1E-03	0.81	0.042	4.1±1.2	3.4±1.6	2.2±2.8
**rs3779788**	19847373	0.98	0.91	0.95	1.2E-03	0.60	0.14	10.4±3.0	8.6±5.4	9.3±4.5
**rs343**	19855067	0.95	0.94	0.95	1.3E-03	0.27	0.16	9.2±2.7	5.7±3.8	12.9±5.3
**rs1059611**	19868843	0.79	0.83	0.81	2.1E-03	0.23	0.11	4.9±1.5	3.3±1.9	8.2±3.5
**rs295**	19860518	0.57	0.73	0.61	3.4E-03	0.61	8.8E-03	3.9±1.3	4.5±1.6	1.6±2.9
**rs3916027**	19869148	0.55	0.69	0.58	4.6E-03	0.77	0.025	3.6±1.2	3.8±1.6	2.2±2.8
**rs264**	19857460	0.87	0.87	0.86	5.3E-03	0.23	0.089	5.1±1.7	4.2±2.3	9.2±3.9
**rs7000460**	19848082	0.74	1.00	0.78	5.5E-03	0.69	0.051	4.4±1.5	3.7±1.8	0.12±4.39
**rs17091872**	19876257	0.81	0.84	0.81	5.7E-03	0.27	0.25	4.4±1.5	2.4±2.0	4.6±3.5
**rs15285**	19868947	0.43	0.67	0.48	5.9E-03	0.70	0.047	3.5±1.2	3.3±1.6	0.30±2.72
**rs3289**	19867472	0.92	1.00	0.92	8.1E-03	0.23	0.22	7.0±2.5	3.8±3.0	10.3±7.5
**rs263**	19857092	0.61	0.86	0.64	8.3E-03	0.092	0.12	3.5±1.3	2.6±1.7	5.6±3.1
**rs2197089**	19870653	0.15	0.41	0.21	9.3E-03	0.72	0.027	4.1±1.5	5.1±2.2	1.3±2.8

P-values for association of TRG with genotype in the total population (p_trg_all) and JHS-AFR-2*_LPL_* (p_trg_afr); SNPs with p<0.0006 are shown in bold. The p-value for interaction (p_int_trg) is for the significance of the genotype×local ancestry term in the linear regression model for the total population (p<0.05 is shown in bold). The percent change in TG level per *LPL* allele is shown with standard error for the total population (effect_all), JHS-AFR-2*_LPL_* (effect_afr) and JHS-EUR-1_2*_LPL_* (effect_eur). The SNP frequencies in the total population (f_all), JHS-AFR-2*_LPL_* (f_afr) and JHS-EUR-2*_LPL_* (f_eur) are also shown, as well as the chromosomal position in bases on chromosome 8.

We successfully genotyped 85 of the 95 tagging SNPs. The strength of the associations for each SNP with TG and HDL-C are shown in Tables S4 and S5 with the top SNPs shown in Tables 4 and 5. In Table 4, three separate p-values per trait are shown for each SNP, representing association across all 3300 individuals (p_all), in the JHS-AFR-2*_LPL_* (p_afr), and for the significance of the genotype-by-ancestry interaction term (p_trg_int). For triglycerides, six of the 85 SNPs were associated at a significance level of p<0.0006 (Bonferroni correction for 85 hypotheses at p<0.05 significance level), including rs328 and all 4 SNPs having r^2^ = 1 with rs328 in JHS-EUR-1_2*_LPL_*: rs1011685 (p = 2.0×10^−5^), rs325 (p = 4.5×10^−5^), rs17482753 (p = 4.9×10^−5^), rs12679834 (p = 2.7×10^−4^). For these 5 SNPs pairwise r^2^ in JHS-AFR-2*_LPL_* ranges from 0.614-1 (Table S3). The strongest association with TRG was in fact for rs10096633 (p = 2.7×10^−6^), where the risk allele for elevated triglycerides is found at a frequency of 0.52 in YRI, and 0.87 in CEU (0.50 in JHS-AFR-2*_LPL_* and 0.82 in JHS-EUR-2*_LPL_*; Table 4). rs10096633 is strongly linked to rs328 in JHS-EUR-2*_LPL_* (r^2^ = 0.79) but not so in JHS-AFR-2*_LPL_* (r^2^ = 0.059).

**Table 5:** Effect size of *LPL* variants on HDL-C levels.

SNP	Position	f_afr	f_eur	f_overall	p_hdl_all	p_int_hdl	effect_all±se	effect_afr±se	effect_eur±se
**rs13702**	19868772	0.43	0.66	0.48	**1.2E-04**	0.73	−1.28±0.33	−1.69±0.44	−0.59±0.69
**rs328**	19864004	0.94	0.86	0.93	6.7E-04	0.23	2.24±0.64	1.57±0.95	1.79±1.10
**rs15285**	19868947	0.43	0.67	0.48	6.9E-04	0.44	1.12±0.32	1.62±0.44	0.16±0.67
**rs325**	19863608	0.94	0.86	0.93	9.5E-04	0.17	−2.15±0.63	−1.38±0.93	−2.13±1.10
**rs343**	19855067	0.95	0.94	0.95	1.2E-03	0.85	−2.43±0.72	−2.80±1.05	−1.77±1.31
**rs10096633**	19875201	0.50	0.82	0.57	1.4E-03	0.71	1.10±0.34	1.20±0.44	0.60±0.76
**rs17482753**	19876926	0.95	0.85	0.94	1.4E-03	0.23	2.19±0.67	1.45±0.99	1.97±1.12
**rs327**	19863816	0.55	0.68	0.59	1.6E-03	0.52	−1.08±0.33	−1.30±0.45	−1.05±0.71
**rs3289**	19867472	0.92	1.00	0.92	2.0E-03	0.58	2.12±0.67	2.64±0.83	1.92±1.79
**rs2197089**	19870653	0.15	0.41	0.21	2.1E-03	0.84	−1.29±0.41	−1.99±0.62	−0.73±0.71
**rs1011685**	19875049	0.95	0.87	0.93	2.4E-03	0.34	2.07±0.66	1.43±0.98	1.68±1.10
**rs3916027**	19869148	0.55	0.69	0.58	3.4E-03	0.41	−0.99±0.33	−1.11±0.45	−1.20±0.68
**rs12679834**	19864713	0.92	0.86	0.91	3.7E-03	0.21	−1.73±0.58	−1.12±0.83	−1.63±1.07
**rs10105606**	19872128	0.26	0.66	0.34	4.5E-03	0.82	−1.03±0.36	−1.48±0.50	−0.66±0.70
**rs331**	19864685	0.56	0.68	0.60	6.1E-03	0.69	−0.92±0.32	−1.13±0.44	−0.71±0.70
**rs1569209**	19874450	1.00	0.90	0.98	7.7E-03	0.78	−3.38±1.24	−7.60±8.22	−2.80±1.38
**rs295**	19860518	0.57	0.73	0.61	8.3E-03	0.64	0.92±0.34	1.19±0.46	0.92±0.72
**rs301**	19861214	0.66	0.74	0.68	8.8E-03	0.82	−0.94±0.35	−1.20±0.47	−0.61±0.73

P-values for association of HDL-C with genotype in the total population (p_hdl_all) and for the significance of the genotype×local ancestry term in the linear regression model (p_int_hdl). SNPs with p<0.0006 are shown in bold. The change in HDL-C level (mg/dL) per *LPL* allele is shown with standard error for the total population (effect_all), JHS-AFR-2*_LPL_* (effect_afr) and JHS-EUR-1_2*_LPL_* (effect_eur). The SNP frequencies in the total population (f_all), JHS-AFR-2*_LPL_* (f_afr) and JHS-EUR-2*_LPL_* (f_eur) are also shown, as well as the chromosomal position in bases.

The rs328 variation leads to a premature stop codon, producing a protein two amino acids short of a full-length product. It has been presumed, based primarily on genetic association data and physiologic studies of *S447X* carriers [29], that this *S447X* variant represents a “gain-of-function” mutation with increased LPL activity. Interestingly, the genotype-local ancestry interaction terms were significant for rs328 (and rs325) (Table 4), indicating a different strength of association on the African and European local ancestry backgrounds. For the African American population as a whole, the estimated effect size was 10.2±2.4% in TG level per rs328 allele. Estimated effect sizes on the African and European local ancestry backgrounds were obtained by repeating the regression modeling using the JHS-AFR*_LPL_* and JHS-EUR*_LPL_* subpopulations. In this analysis, the effect size per rs328 allele was 4.5±3.4% on the African local ancestry background (Table 4, p = 0.22) and 18.6±4.5% in the subgroup with one or more European *LPL* chromosomes. The results are similar for the highly correlated rs325, rs17482753, rs1011685 and rs12679834 SNPs, with all showing a significant genotype-local ancestry interaction. The significant dependence of effect size on local ancestral background suggests that the effect seen in association studies in European populations is not predominantly mediated by rs328, but arises either from linkage of rs328 and other highly correlated SNPs to some other causal variant, or from the aggregate effect of multiple tightly linked causal variants. The effect size for rs10096633 is more comparable in the three populations: (6.2±1.2%, 4.5±1.6%, and 6.6±3.1% for total, JHS-AFR-2*_LPL_*, and JHS-EUR-1_2*_LPL_*, respectively; Table 4). Although some SNPs show a marked difference in effect size between local ancestry backgrounds, the overall difference in genotype regression coefficients for the 85 SNPs in JHS-AFR-2*_LPL_* and JHS-EUR-1_2*_LPL_* falls short of statistical significance (p = 0.07, Wilcoxon signed-ranks test).

To determine if a multi-SNP model could better explain the variability in TG levels, we performed stepwise linear regression combined with ANOVA (see Methods) for the 100 sets of unrelated JHS members to identify the top independent SNP signals. The most frequently observed model included rs10096633, rs1031045, rs3779788, rs11995036. A list of the top models is shown in Table S6a.

Previous studies have established that LPL activity also influences HDL-C levels, presumably by producing remnants of triglyceride-rich lipoproteins, which can be used for HDL assembly [30]. The *LPL* rs328 SNP and its proxies have also been shown to be directly associated with HDL-C levels in populations of European descent [14]. We therefore looked for association of our fine-mapping *LPL* SNPs with HDL-C levels (Table 5 for top SNPs, Table S5 for all SNPs) and found association of one SNP with HDL-C at the p<0.0006 significance level: rs13702 (r^2^ = 0.72 with rs10096633 in JHS-AFR-2*_LPL_*, and allele frequency of 0.43 in JHS-AFR-2*_LPL_*). The top model of independent signals for HDL-C is rs13702, rs3289, rs343, rs10283151, rs2197089, rs6651471, rs9644636 (Table S6b).

The hypothesis of a differential effect size for rs328 on different local ancestry backgrounds may be further evaluated using individuals who are heterozygotes for both genotype and local ancestry background. Presumably genotype heterozygotes might show different TG levels depending on whether the allele for higher TG resides on the African or European local ancestry background at *LPL*. However, when we used ANCESTRYMAP to output phased genotypes at rs328 (data not shown), the number of individuals for whom we could generate confident estimates of phase and local ancestry was insufficient for comparison.

In conclusion, using local ancestry estimates at the *LPL* locus to minimize confounding by population stratification, we have identified novel common variants within the *LPL* gene that are moderately differentiated in frequency between African and European Americans, and strongly associated with TG and HDL-C levels. Furthermore, analysis of differential local ancestry backgrounds suggests that the rs328 SNP explains at most a modest amount of the TG variation at the *LPL* locus seen in association studies in populations of European descent.

### Genetic Association Analysis at Validated Lipid and Cholesterol Loci Confirms the Importance of Local Ancestry Estimates

To explore whether the use of local ancestry estimates informs the analysis of other SNPs known to be strongly associated with lipid and cholesterol traits, we genotyped 12 index SNPs (from 10 genes) that met genome-wide significance in recent lipid/cholesterol GWAS. These have not yet been evaluated for association in a large African-American population such as JHS. Table 6 shows the overall p-value and a comparison of effect sizes in the JHS-AFR-2 (n = 1727–1945, depending on the gene) and JHS-EUR-1_2 (n = 540–728) subgroups at the locus of interest, and the p-value for interaction of local ancestry with genotype. We replicate the original association at p<0.05 for 7 of the 12 SNPs (excluding rs328). The failure to replicate for the others may be a consequence of reduced power due to sample size (the original meta-analyses exceeded 8000 individuals) or a lower risk allele frequency arising from genetic drift. There may also be fundamental differences in effect size between European and African Americans, reflecting unidentified gene×gene or gene×environment interactions.

**Table 6:** Comparison of association of validated SNPs with lipid and cholesterol phenotypes in JHS, JHS-AFR-2*_LPL_* and JHS-EUR-2*_LPL_*.

Trait	Gene	SNP	Type	p_all	p_all_int	effect_all±se	effect_afr±se	effect_eur±se	f_afr	f_eur	f_all
HDL-C	*LIPC*	rs1800588	Upstream	0.10	0.53	0.56±0.34	0.55±0.34	1.3±0.8	0.50	0.58	0.50
HDL-C	*LIPG*	rs2156552	Intergenic	**0.011**	0.84	−2.5±0.9	−3.5±2.2	−2.3±1.3	0.97	0.91	0.97
HDL-C	*CETP*	rs3764261	Downstream	**2.7E-09**	0.86	−2.1±0.3	−2.0±0.3	−2.1±0.8	0.67	0.67	0.67
HDL-C	*ABCA1*	rs3890182	Intronic	0.14	0.06	0.75±0.50	0.85±0.51	−1.2±1.1	0.88	0.86	0.88
HDL-C	*LIPC*	rs4775041	Intergenic	**7.2E-03**	0.21	−1.4±0.5	−1.1±0.5	−1.7±0.9	0.88	0.90	0.88
LDL-C	*PCSK9*	rs11591147	Coding (Non-Syn)	**1.2E-03**	0.12	−31.4±9.6	NA	−24.9±12.1	1.00	1.00	1.00
LDL-C	*HMGCR*	rs12654264	Intronic	0.085	0.54	1.9±1.1	1.4±1.2	3.4±2.2	0.68	0.68	0.68
LDL-C	*LDLR*	rs6511720	Intronic	**1.4E-06**	0.79	−7.0±1.4	−7.0±1.5	−7.1±3.1	0.86	0.87	0.86
LDL-C	*ApoB*	rs693	Coding (Syn)	0.21	0.17	−1.6±1.3	−0.78±1.58	−3.7±2.3	0.79	0.67	0.78
TG	*ApoA5*	rs3135506	Coding (Non-Syn)	**1.6E-06**	0.39	13.6±2.6	11.9±0.9	14.8±7.0	0.95	0.98	0.94
TG	*ApoA5*	rs662799	5′-UTR	0.069	**0.03**	3.5±1.9	1.1±1.0	21.5±5.5	0.88	0.86	0.88
TG	*GCKR*	rs780094	Intronic	**3.8E-04**	0.56	6.4±1.7	5.5±1.1	8.4±3.2	0.83	0.82	0.82
TG	*LPL*	rs328	Nonsense	**7.1E-05**	**0.05**	10.2±2.4	4.5±3.4	18.6±4.5	0.94	0.86	0.93

P-values for association of TG, LDL-C or HDL-C with genotype in the total population (p_all) and p-value for interaction (p_all_int) are shown (p≤0.05 is shown in bold). The percent change in TG or change in HDL-C or LDL-C level (mg/dL) per allele is shown with standard error for the total population (effect_all), JHS-AFR-2*_LPL_* (effect_afr) and JHS-EUR-1_2*_LPL_* (effect_eur). The SNP frequencies in the total population (f_all), JHS-AFR-2*_LPL_* (f_afr) and JHS-EUR-2*_LPL_* (f_eur) are also shown.

Just as with *LPL*, we can compare effect sizes in JHS-AFR with JHS-EUR to look for any systematic variation with ancestral background. Given that individuals in the cohort are likely to share similar environments (independent of local ancestry at the site of the tested variant), this type of internal comparison of effect sizes is superior to comparing effects across different ethnic populations, where multiple confounding factors may play a role. We see that for 10 of the 12 index SNPs (excluding rs11591147, which is fixed in YRI, but including rs328), the magnitude of the effect is stronger in the European local ancestry subgroup than in the African local ancestry subgroup, which is significant at p = 0.034 by the Wilcoxon signed rank test (using standardized residuals as the predicted variable). We note that some of the differences are very small; nonetheless, this estimate is probably conservative, as our JHS-EUR-1_2 subgroups include many individuals with one allele of African local ancestry at *LPL*. The systematic difference in effect size suggests that the majority of these index SNPs are markers for the major causal SNP(s), with weaker SNP-SNP correlations seen in the ancestral West African population than in the ancestral European population, leading to smaller effect sizes.

Some examples are illustrative. rs3135506, which encodes for a S19W mutation in the endoplasmic reticulum signal peptide of *ApoA5* [31] is likely responsible for the majority of the effect seen in association studies in both populations, given its similar effect sizes in JHS-AFR-2 *_ApoA5_* (11.9±0.9 mg/dL) and JHS-EUR-1_2 *_ApoA5_* (14.8±7.0 mg/dL). Further, it is not likely that there are other major causal variants linked to rs3135506 in one population but not the other.

The intronic rs780094 variant in *GCKR* is significantly associated with TG in JHS (p = 0.00038). The effect size in JHS-AFR*_GCKR_* (5.5±1.1% per allele) is smaller than that seen in JHS-EUR*_GCKR_* (8.4±3.2% per allele), although the p-value for interaction is not significant (p = 0.56). This observation can be explained by considering LD patterns in the respective HapMap populations with respect to the likely causal allele, rs1260326, which encodes a leucine to proline change at amino acid 446 [16]. The r^2^ value of rs780094 with rs1260326 is 0.93 in CEU but only 0.42 in YRI. This modest difference can explain the similarly modest difference in effect sizes between JHS-EUR-2*_GCKR_* and JHS-AFR-2*_GCKR_*, although given our relatively small sample size, we cannot exclude the contribution of chance variation.

Local ancestry analysis reveals a similar pattern for most of the other SNPs tested, with significant overall association and with effect size in JHS-EUR exceeding that in JHS-AFR. A more marked variation in effect size with ancestry is seen for rs662799 in *ApoA5*. This SNP, found in the 5′ UTR of the *ApoA5* gene, has a strong association in Willer et al [16] with an estimated effect size per allele of 16.9 mg/dl. An even stronger effect size is seen in JHS-EUR*_ApoA5_* (21.5±5.5% per allele) but the effect is negligible in JHS-AFR*_ApoA5_* (1.1±1.0% per allele), leading to a significant p-value for interaction (p = 0.03). rs662799 and rs328 (*LPL*) are thus the two SNPs that show a sufficiently marked difference in effect sizes between local ancestry subgroups to have a significant interaction effect.

## Discussion

We have demonstrated that local ancestry-based analysis in admixed populations such as African Americans adds novel insights beyond studies of genetic determinants of disease performed in populations of European descent. We used precise individual ancestry estimates to show a highly significant association of individual African/European ancestry with serum TG and HDL-C, suggesting strongly that genetic factors account for at least some of the epidemiologically observed interethnic differences. However, using local ancestry estimates in the context of whole-genome admixture mapping for TG, HDL-C, and LDL-C, we did not identify any genes with strong contributions to these trends. Thus, although it is likely that there are genetic variants whose frequency differences contribute to interethnic variability in lipid profile, these appear to have relatively modest effects. This is in keeping with our prior work on hypertension [9], and with the generally modest effects of lipid profile variants seen in recent GWAS/meta-analysis studies [14]–[18].

Analysis of the association of local ancestry with TG/HDL-C/LDL-C at validated lipid profile loci led to prioritization of *LPL* for fine-mapping. Overall, we identified rs10096633 as a credible candidate for variation in TG levels, and rs13702 as a candidate for variation in HDL-C. Remarkably, analysis within the homozygote African local ancestry background showed that the extensively studied rs328 variant, which encodes a premature stop codon in *LPL*, is directly responsible for at most a modest amount of variation in TG levels.

The *S447X* premature stop codon, caused by the rs328 variant in *LPL*, has a contentious history as a putative gain-of-function mutation, with considerable debate as to whether it demonstrates increased lipolytic activity [29],[32]. Interestingly, it has also been associated with a reduced risk of cardiovascular disease, including myocardial infarction [33]. Largely on the basis of the observed beneficial associations of the *S447X* variant in population studies, therapeutic trials with viral delivery of the *S447X LPL* transgene have been conducted in animal models [34],[35], and safety trials in lipoprotein lipase-deficient patients have also begun [36]. Our findings suggest that the *S447X* variant may not be the major causal SNP within *LPL* that influences TG levels, and that it is primarily a marker for the causal variant(s) in European-derived populations. This finding—taking advantage of the unique short range linkage disequilibrium in chromosomes of African ancestry—emphasizes the challenges of assigning causality based on genetic association, and is cautionary for the marketing of genetic marker-based tests for disease risk assessment. When these tests are based on any variant other than the causal allele, utility may not extend to ethnic groups differing from the original study population. Prognostication in African Americans based on a SNP such as rs328 would require knowledge of *LPL* local ancestry for each individual while, for a SNP like rs10096633, with more uniform effect size, this information would not be required.

Although SNPs with much weaker effects on one ancestral background may just be non-functional markers, situations could also exist where they could be causal but still have differential effects. The most plausible scenario might be that multiple functional variants comprise a single haplotype more frequently in one subpopulation than in the other. This might be the case more often in European populations (and on local European ancestral background in admixed populations), where LD extends over longer distances, resulting in systematically lower effect sizes on African local ancestry backgrounds for SNPs identified in GWAS studies of European-derived populations. Even in the case where there is only a single causal variant being tested, gene-gene epistatic effects from variants at other loci that are at different frequencies in the two subpopulations could influence effect size. Finally, environmental influences on effect size may also differ by ancestral background.

In JHS-EUR-1*_LPL_* (19% of our population), the effect sizes for rs328 (minor allele frequency of 12.5% in CEU) and closely correlated SNPs are large, ranging from 18–22% of variation in TG levels per risk allele, and accounting for almost 4% of the residual trait variance. This effect is comparable to or exceeds the effect of rs328 (or its proxies) in populations of European descent, which has been estimated at 14 mg/dL [16] or 13.8% [17] per allele. The strongest effect size for *LPL* variants in JHS-AFR-2*_LPL_* is less, at 6% (for rs10096633 and rs13702). Since we tagged the *LPL* gene densely this raises the question of why we failed to find a variant with a large effect size on this African local ancestry background. Possible explanations include that the rs328 variant is in LD with multiple variants affecting TG levels in the European local ancestry background, that epistatic effects of other frequency-differentiated SNPs interact with that of rs328, that the true risk variant(s) may lie outside our tagging boundaries, or that a strong risk allele in LD with rs328 in Europeans is at very low frequency in the West African population.

We extended our local ancestry based association analysis to previously validated TG/cholesterol loci, and found replication for 7 of 12 loci at p<0.05. However, we observed an overall trend of weaker effect size in JHS-AFR than JHS-EUR, with some marked differences such as rs662799 in *ApoA5*, suggesting that many of these “index” SNPs are merely tagging causal alleles, with the effect size depending on the correlation between the index SNP and the causal SNP(s). Again, this has clear implications for the use of SNPs identified in one ethnic population as markers of disease risk in other groups with differing demographic history.

In conclusion, we have developed a local-ancestry based approach to genetic association analysis in admixed populations, and using it, we have identified several variants in the *LPL* gene that contribute to African/European American differences in lipid profiles. The differing patterns of linkage disequilibrium on different local ancestry backgrounds have allowed us to explore plausibility of causality for established variants. We have also laid the foundation for future studies to identify variants that would be suitable as risk markers in African American populations. Finally, our work highlights some of the challenges and opportunities that derive from extending the results of genetic association analyses across ethnic groups, and admixed groups in particular.

## Materials and Methods

### Statistical Analysis

Linear and logistic regression analyses were used to evaluate association of global ancestry with lipid and cholesterol phenotypes. For linear regression, individuals were ranked in terms of increasing quantitative trait, and the top and bottom 0.5% of individuals were eliminated from further analysis as these extreme phenotypes are thought to be more likely attributable to monogenic disorders. For the LDL-C analyses, all individuals on cholesterol-lowering medication were also eliminated, as this trait is particularly sensitive to therapy. For the logistic regression analysis, “cases” and “controls” were selected as the top and bottom quintile of the distribution for each trait. A logistic regression model was then developed to predict case or control status for each trait including age, age^2^, BMI, BMI^2^, gender, type 2 diabetes mellitus and smoking status as possible covariates, with covariates selected for the model by stepwise forward regression. The significance of association of case/control status and global African/European ancestry was assessed using nested logistic regression models and the likelihood ratio test [37].

We randomly selected only one member of each family to be included in all genotypic analyses. To minimize bias, we generated 100 overlapping sets of 3300 unrelated individuals and conducted all statistical analyses on each set, averaging the results.

For linear regression, triglyceride values were log transformed. The individual's multivariable-adjusted lipid residual was used as the phenotype in phenotype association analyses with global ancestry, local ancestry, and genotype. For genotype-phenotype association analyses, we assumed an additive model of inheritance and tested for the strength of association by ANOVA [38] with nested linear regression models, which included local ancestry, local ancestry+genotype, and local ancestry+genotype+genotype×local ancestry interaction. The genotype-local ancestry interaction term was computed as a product of the local-ancestry term and SNP genotype, and is a continuous variable ranging from of 0 to 2. We also conducted separate linear regression in a subset of 1860 individuals with a >95% probability of two African ancestry alleles at the *LPL* locus and in a subset of 728 individuals with a high (>95%) probability of 1 or more European ancestral alleles (local ancestry >48% European ancestry). For the subset of individuals with homozygous African local ancestry, we tested the association of the multivariable-adjusted lipid residual against the SNP genotype in an additive model of inheritance. Effect sizes were estimated by evaluating what effect a unit change in genotype would have on the predicted value of the trait.

Multi-SNP models were identified with stepwise linear regression and the *anova* function in R. For each of the 100 sets of 3300 individuals, the top SNP associated with the phenotype residual of interest was identified and added to the regression model. The remaining SNPs were then each tested by comparing the model with and without the SNP by ANOVA and the most significant SNP included if the p-value for comparing was <0.05. This process was continued for each of the 100 sets of individuals until no additional SNP improved the model at the p<0.05 threshold.

For each SNP that retained significance after a Bonferroni correction, we re-estimated local ancestry at *LPL* by forcing it into the set of markers used to estimate local ancestry (see below), so that the joint distribution of ancestry and genotype could be appropriately tested for association.

Estimation of allele frequencies and r^2^ for SNPs was performed using the R GeneticsBase package. All statistical analyses were performed using R (2.7.0).

### Selection of Case and Control Samples

The samples in this study (n = 4605) were all self-identified African Americans in the Jackson Heart Study [21]. Between Sept. 2000 and March 2004, 5,302 African Americans were recruited from three counties, Hinds, Rankin, and Madison, which comprise the Jackson, MS metropolitan area. Unrelated JHS participants were drawn from three sources in roughly equal numbers: (1) former ARIC participants; (2) participants selected randomly from a commercial residential listing; and (3) a constrained volunteer sample for which demographic cells for recruitment were designed to mirror the overall target population.

For each trait (TG, LDL-C, HDL-C), the linear regression residual was calculated for each individual with either a minimally adjusted model, using age, age^2^, and gender as possible covariates, or a fully adjusted model with age, age^2^, BMI, BMI^2^, gender, type 2 diabetes mellitus and smoking status as possible covariates. Individuals were ranked by regression residual, and the top and bottom quintiles of individuals were selected to be cases and controls respectively for the admixture scans.

For each trait and each regression model (3 traits×2 regression models), we selected only one member of each family to be included in the admixture run. This was selected to be the individual with either the highest regression residual for that run or the lowest regression residual for that run. A separate run was performed for each combination, leading to a total of 12 admixture scans (Table S1).

### Admixture Mapping and Markov Chain Monte Carlo Data Analysis for Inference of Ancestry and Testing of Disease Association

The ANCESTRYMAP software[10] was used for all analyses. The program generates local ancestry estimates by integrating information from a panel of densely spaced markers differentiated in frequency between African and European populations. The 4464 JHS individuals were genotyped on one of two panels of markers informative for West African vs. European ancestry [8]: 976 were genotyped on an older “Phase 2” Panel of 1536 markers, and 3488 were genotyped on an updated “Phase 3” Panel of 1536 markers. After quality checks, 1408 SNPs in the “Phase 2” Panel and 1447 SNPs in the “Phase 3” Panel were used for subsequent analyses. The LOD score for association, defined as the log ratio of the likelihood of the data under a disease model divided by the likelihood of the data under no disease model, was evaluated at equally spaced points across the genome. At each of these points, the disease likelihood was evaluated with a multiplicative risk model, with risk of disease integrated over the inheritance of 0, 1, and 2 copies of an African ancestral allele. Being based on Bayesian statistics, the ANCESTRYMAP software requires specification of a prior distribution of risk models; we used a range of ten risk models from 1.5-fold increased risk due to inheritance of one African ancestral allele to 1.5-fold increased risk due to inheritance of one European allele, with cases, controls, or both used in the analysis. For each point in the genome, we averaged the Bayes factors generated for each risk model, with the LOD score corresponding to the log-base-10 of this number.

### Frequency Estimates from the Ancestral Populations

Frequency estimates for each of the SNPs in Africans and Europeans, were obtained with previously published data [6],[39] and data from the International HapMap Project [40]. These samples provided a Bayesian prior distribution for the parental population allele frequencies as described in reference [10].

### Construction of Exclusion Map

To obtain credible intervals for increased risk due to African ancestry across the genome, we repeated the procedure described in [9]. We ran ANCESTRYMAP repeatedly for 65 separate disease risk models (0.40, 0.42, 0.44, 0.46 …, 1.66, 1.68 and 1.70-fold increased risk due to one European allele), and searching for the maximum likelihood risk model. We evaluated LOD scores at equally spaced points across the genome, and for each point we averaged the LOD scores for the four runs of each lipid or cholesterol trait. The 95% credible intervals for increased risk due to African (or European) ancestry were obtained by a likelihood ratio test, with the interval including all risk models for which the log-base-10 of the likelihood of the disease model was within 0.883 of the maximum. We also computed an exclusion map for each trait for a 1.5-fold increased risk of Case status with inheritance of one copy of the African (or European) local ancestral allele. We evaluated the Cases-only LOD score at 3,622 equally spaced points across the genome, and excluded points with LOD scores <−2. The percentage of points excluded was 84.2% (increased risk due to African ancestry) and 88.5% (increased risk due to European ancestry) for TG; 82.6% and 91.6% for HDL-C; and 81.8% and 88.9% for LDL-C.

### Evaluation of Association of Local Ancestry at Established Loci with Lipid Traits

For each of the previously validated lipid and cholesterol loci, we identified the genetic position of the most strongly associated SNP using NCBI Build 35 of the public genome reference sequence (http://genome.ucsc.edu) and selected the closest marker (among equally-spaced markers across the genome, see above) to estimate the local ancestry at that locus. Local ancestry was tested for association with lipid phenotypes in a linear regression model as detailed above.

### Online Resources

http://genepath.med.harvard.edu/˜reich for our ANCESTRYMAP software.

### Accession Numbers; Entrez ID for Discussed Genes

APOE – 348; HMGCR – 3156; LDLR – 3949; MVK – 4598; NCAN; PCSK9 – 255738; SORT1 – 6272; ABCA1 – 19; APOA5 – 116519; CETP – 1071; GALNT2 – 2590; LIPC – 3990; LIPG – 9388; LPL – 4023; MLXIPL – 51085; TRIB1 – 10221; ANGPTL3 – 27329; APOB 338; GCKR 2646; NCAN – 1463.

## Supporting Information

Figure S1 Average Cases-Only LOD Scores Across the Genome for a) TG, b) HDL-C, and c) LDL-C. The risk model specified is for a 1.5-fold increased risk of Case status with inheritance of a single copy of the i) African or ii) European ancestral allele.
(0.55 MB DOC)

Table S1 Summary of Admixture Scans for Lipid and Cholesterol Traits. For each trait, 4 scans were performed, varying the predictors used in the regression model to select cases and controls, and the method of selecting unrelated individuals. The genome-wide LOD score and top scores for the Cases only and Case-Control statistics are shown. The thresholds of a significant association for the genome-wide and Cases only LOD scores are 2.0 and 5.0, respectively.
(0.04 MB DOC)

Table S2 Proportion of genome excluded as contributing to differential risk for triglycerides and cholesterol phenotypes comparing African and European Americans.
(0.03 MB DOC)

Table S3 Extent of pairwise linkage disequilibrium measured by r^2^ for select SNPs in JHS-AFR-2*_LPL_* and JHS-EUR-2*_LPL_*.
(0.13 MB DOC)

Table S4 Effect size of *LPL* variants on TG levels. P-values for association of TRG with genotype in the total population (p_trg_all) and JHS-AFR-2*_LPL_* (p_trg_afr); SNPs with p<0.0006 are shown in bold. The p-value for interaction is for the significance of the genotype×local ancestry term in the linear regression model for the total population (p<0.05 is shown in bold). The percent change in TG level per *LPL* allele is shown with standard error (SE) for the total population (effect_all), JHS-AFR-2*_LPL_* (effect_afr) and JHS-EUR-1_2*_LPL_* (effect_eur). The SNP frequencies in the total population (f_all), JHS-AFR-2*_LPL_* (f_afr) and JHS-EUR-2*_LPL_* (f_eur) are also shown, as well as the chromosomal position in bases on chromosome 8.
(0.19 MB DOC)

Table S5 Effect size of *LPL* variants on HDL-C levels. P-values for association of HDL-C with genotype in the total population (p_hdl_all) and for the significance of the genotype×local ancestry term in the linear regression model. SNPs with p<0.0006 are shown in bold. The change in HDL-C level (mg/dL) per *LPL* allele is shown with standard error for the total population (effect_all), JHS-AFR-2*_LPL_* (effect_afr) and JHS-EUR-1_2*_LPL_* (effect_eur). The SNP frequencies in the total population (f_all), JHS-AFR-2*_LPL_* (f_afr) and JHS-EUR-2*_LPL_* (f_eur) are also shown, as well as the chromosomal position in bases on chromosome 8.
(0.18 MB DOC)

Table S6 Multi-SNP models derived using nested regression models and ANOVA for a) TG and b) HDL-C. The most frequent solutions are shown for the 100 runs performed (one run per set of 3300 unrelated individuals).
(0.04 MB DOC)
