# Supplementary material for: Genetic Differences between the Determinants of Lipid Profile Phenotypes in African and European Americans: The Jackson Heart Study
Source: PLoS Genet. 2009 Jan 16;5(1):e1000342. doi: 10.1371/journal.pgen.1000342 (PMC2613537; doi:10.1371/journal.pgen.1000342)

**Supplementary Figure 1: Average Cases-Only LOD Scores Across the Genome for a) TG, b)HDL-C, and c) LDL-C.** The risk model specified is for a 1.5-fold increased risk of Case status with inheritance of a single copy of the i) African or ii) European ancestral allele.

a) TG

i) Cases-only LOD score for a risk model of 1.5-fold increased risk with inheritance of the African ancestral allele


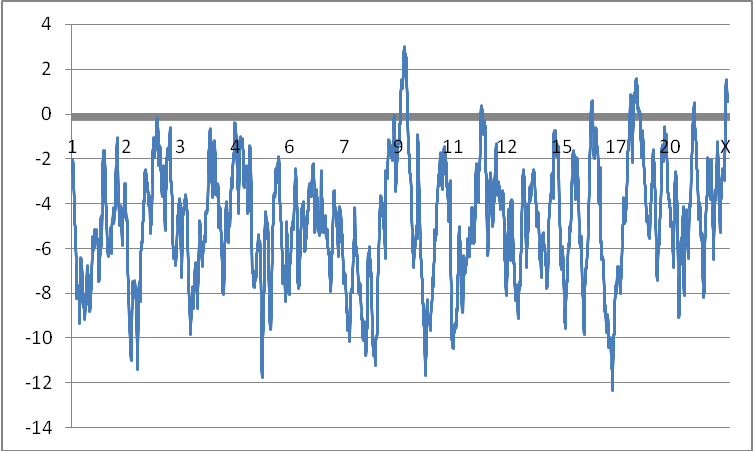


ii) Cases-only LOD score for a risk model of 1.5-fold increased risk with inheritance of the European ancestral allele


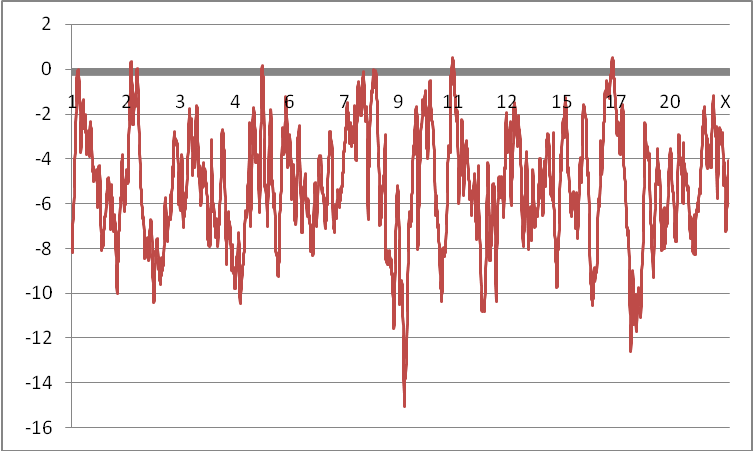


b) HDL-C

i) Cases-only LOD score for a risk model of 1.5-fold increased risk with inheritance of the African ancestral allele


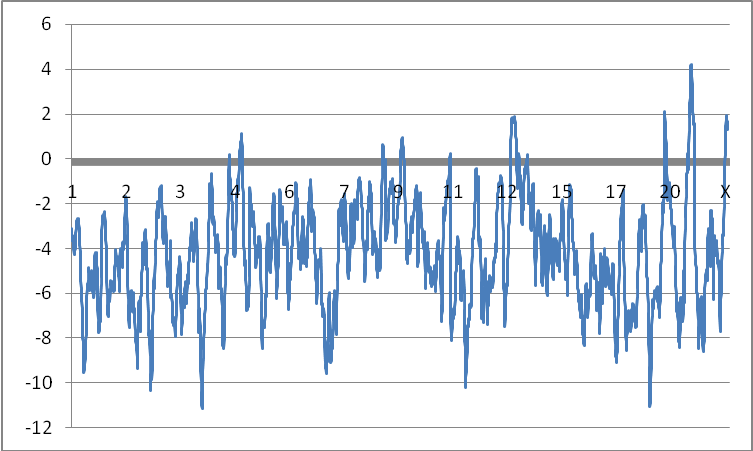


ii) Cases-only LOD score for a risk model of 1.5-fold increased risk with inheritance of the European ancestral allele


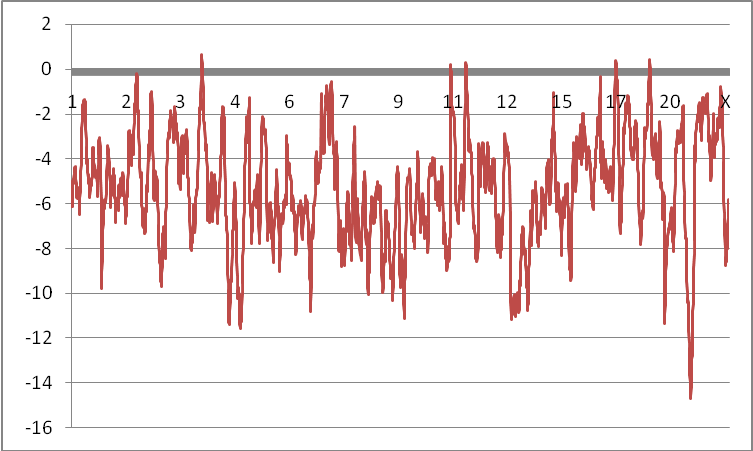


c) LDL-C

i) Cases-only LOD score for a risk model of 1.5-fold increased risk with inheritance of the African ancestral allele


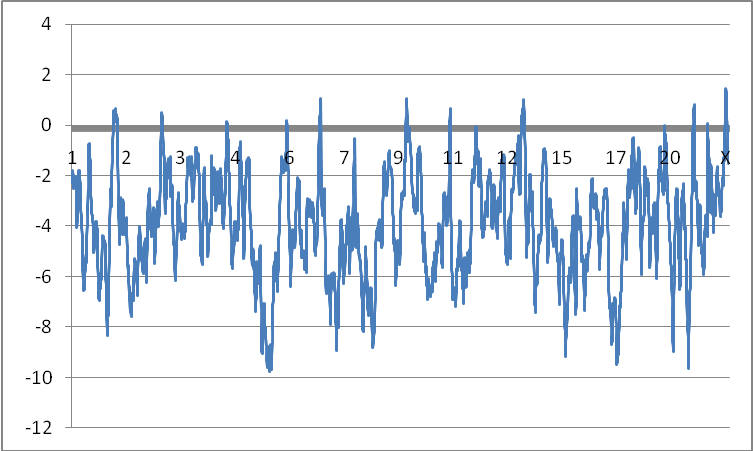


ii) Cases-only LOD score for a risk model of 1.5-fold increased risk with inheritance of the European ancestral allele


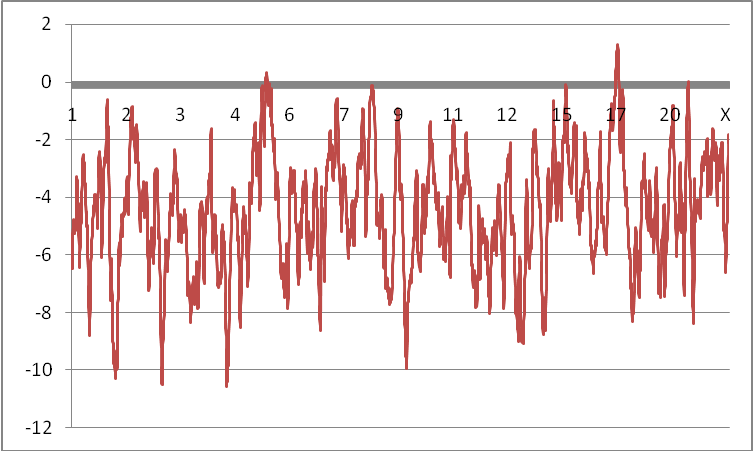

Supplement: Figure S1 — Average Cases-Only LOD Scores Across the Genome for a) TG, b) HDL-C, and c) LDL-C. The risk model specified is for a 1.5-fold increased risk of Case status with inheritance of a single copy of the i) African or ii) European ancestral allele. (0.55 MB DOC) [file pgen.1000342.s001.doc]
