# Supplementary material for: Genetic Differences between the Determinants of Lipid Profile Phenotypes in African and European Americans: The Jackson Heart Study
Source: PLoS Genet. 2009 Jan 16;5(1):e1000342. doi: 10.1371/journal.pgen.1000342 (PMC2613537; doi:10.1371/journal.pgen.1000342)
Supplement: Table S1 — Summary of Admixture Scans for Lipid and Cholesterol Traits. For each trait, 4 scans were performed, varying the predictors used in the regression model to select cases and controls, and the method of selecting unrelated individuals. The genome-wide LOD score and top scores for the Cases only and Case-Control statistics are shown. The thresholds of a significant association for the genome-wide and Cases only LOD scores are 2.0 and 5.0, respectively. (0.04 MB DOC) [file pgen.1000342.s002.doc]

**Supplementary Table 1: Summary of Admixture Scans for Lipid and Cholesterol Traits.** For each trait, 4 scans were performed, varying the predictors used in the regression model to select cases and controls, and the method of selecting unrelated individuals. The genome-wide LOD score and top scores for the Cases only and Case-Control statistics are shown. The thresholds of a significant association for the genome-wide and Cases only LOD scores are 2.0 and 5.0, respectively.

| **Trait** | **Covariates Adjusted For** | **Regression Residual of Included Family Member** | **Genome-Wide LOD Score** | **Top LOD Score : Cases Only Statistic** | **Top Score: Case-Control Statistic** |
| --- | --- | --- | --- | --- | --- |
| TG | Full^1^ | Highest | 0.063 | 2.228 | -3.426 |
| TG | Minimal^2^ | Highest | 0.093 | 1.983 | -3.718 |
| TG | Full^1^ | Lowest | -0.174 | 1.767 | 3.165 |
| TG | Minimal^2^ | Lowest | 0.041 | 1.922 | 3.630 |
| HDL-C | Full^3^ | Highest | 0.489 | 3.125 | -3.128 |
| HDL-C | Minimal^4^ | Highest | 0.351 | 3.203 | -3.854 |
| HDL-C | Full^3^ | Lowest | 0.581 | 2.920 | -3.395 |
| HDL-C | Minimal^4^ | Lowest | 0.614 | 3.316 | -4.459 |
| LDL-C | Full^1^ | Highest | -0.212 | 1.890 | -3.114 |
| LDL-C | Minimal^2^ | Highest | -0.012 | 1.846 | -3.001 |
| LDL-C | Full^1^ | Lowest | -0.241 | 1.823 | -3.287 |
| LDL-C | Minimal^2^ | Lowest | 0.001 | 1.872 | 3.486 |

^1^Adjusted for age, age^2^, gender, bmi, bmi^2^, dm2, smoking

^2^Adjusted for age, age^2^, gender

^3^Adjusted for gender, bmi, bmi^2^, dm2

^4^Adjusted for gender
