# Supplementary material for: Genetic Differences between the Determinants of Lipid Profile Phenotypes in African and European Americans: The Jackson Heart Study
Source: PLoS Genet. 2009 Jan 16;5(1):e1000342. doi: 10.1371/journal.pgen.1000342 (PMC2613537; doi:10.1371/journal.pgen.1000342)
Supplement: Table S2 — Proportion of genome excluded as contributing to differential risk for triglycerides and cholesterol phenotypes comparing African and European Americans. (0.03 MB DOC) [file pgen.1000342.s003.doc]

**Supplementary Table 2: Proportion of genome excluded as contributing to differential risk for triglycerides and cholesterol phenotypes comparing African and European Americans.**

| Factor by which AFRICAN ancestry increases risk at this locus compared with European ancestry | Percent of genome excluded as having this risk or more at P<0.05 | | | Factor by which European ancestry increases risk at this locus compared with African ancestry | Percent of genome excluded as having this risk or more at P<0.05 | | |
| --- | --- | --- | --- | --- | --- | --- | --- |
|  | TG | HDL-C | LDL-C |  | TG | HDL-C | LDL-C |
| 1.0 | 5 | 5 | 5 | 1.0 | 5 | 5 | 5 |
| 1.1 | 27 | 21 | 18 | 1.1 | 24 | 24 | 19 |
| 1.2 | 56 | 50 | 48 | 1.2 | 55 | 59 | 51 |
| 1.3 | 79 | 77 | 76 | 1.3 | 82 | 86 | 82 |
| 1.4 | 93 | 91 | 92 | 1.4 | 96 | 98 | 96 |
| 1.5 | 97 | 96 | 97 | 1.5 | 100 | 100 | 100 |

Note: The percentage of the genome where the null hypothesis (relative risk due to ancestry = 1) is excluded is about 5% for all scenarios, as expected using a P<0.05 significance cutoff.
