# Supplementary material for: Genetic Differences between the Determinants of Lipid Profile Phenotypes in African and European Americans: The Jackson Heart Study
Source: PLoS Genet. 2009 Jan 16;5(1):e1000342. doi: 10.1371/journal.pgen.1000342 (PMC2613537; doi:10.1371/journal.pgen.1000342)
Supplement: Table S3 — Extent of pairwise linkage disequilibrium measured by r2 for select SNPs in JHS-AFR-2LPL and JHS-EUR-2LPL. (0.13 MB DOC) [file pgen.1000342.s004.doc]

**Supplementary Table 3:** **Extent of pairwise linkage disequilibrium measured by r^2^ for select SNPs in JHS-AFR-2*_LPL_* and JHS-EUR-2*_LPL_.***

a) JHS-AFR-2*_LPL_*

| **SNP2** | **SNP3** | **SNP4** | **SNP5** | **SNP6** | **SNP7** | **SNP8** | **SNP9** | **SNP10** | **SNP11** | **SNP12** | **SNP13** | **SNP14** | **SNP15** | **SNP16** | **SNP17** | **SNP18** | **SNP19** | **AFR** |
| --- | --- | --- | --- | --- | --- | --- | --- | --- | --- | --- | --- | --- | --- | --- | --- | --- | --- | --- |
| 0.048 | 0.001 | 0.008 | 0.003 | 0 | 0.002 | 0.046 | 0.002 | 0.003 | 0.001 | 0.061 | 0 | 0.007 | 0 | 0.003 | 0.045 | 0 | 0.002 | **SNP1** |
|  | 0.018 | 0.13 | 0.016 | 0.03 | 0.009 | 0.004 | 0.008 | 0 | 0.046 | 0.026 | 0.01 | 0.099 | 0 | 0.018 | 0.039 | 0.011 | 0.018 | **SNP2** |
|  |  | 0.004 | 0.248 | 0.001 | 0.143 | 0.008 | 0.15 | 0.114 | 0 | 0.003 | 0.001 | 0.005 | 0.025 | 0.174 | 0.003 | 0 | 0.174 | **SNP3** |
|  |  |  | 0.008 | 0.002 | 0.001 | 0 | 0.001 | 0.001 | 0 | 0.039 | 0.001 | 0.024 | 0 | 0.009 | 0.062 | 0.017 | 0.009 | **SNP4** |
|  |  |  |  | 0.002 | 0.591 | 0.054 | 0.605 | 0.436 | 0.001 | 0.028 | 0.001 | 0.009 | 0.012 | 0.668 | 0.035 | 0 | 0.661 | **SNP5** |
|  |  |  |  |  | 0.003 | 0.013 | 0.003 | 0.002 | 0.003 | 0.025 | 0.279 | 0.125 | 0 | 0.002 | 0.034 | 0 | 0.002 | **SNP6** |
|  |  |  |  |  |  | 0.081 | 0.985 | 0.726 | 0.004 | 0.045 | 0.001 | 0.012 | 0.009 | 0.838 | 0.061 | 0 | 0.841 | **SNP7** |
|  |  |  |  |  |  |  | 0.079 | 0.108 | 0.066 | 0.499 | 0.012 | 0.127 | 0.001 | 0.067 | 0.328 | 0.009 | 0.069 | **SNP8** |
|  |  |  |  |  |  |  |  | 0.724 | 0.004 | 0.044 | 0.001 | 0.012 | 0.009 | 0.854 | 0.059 | 0 | 0.852 | **SNP9** |
|  |  |  |  |  |  |  |  |  | 0.006 | 0.058 | 0.001 | 0.014 | 0.006 | 0.614 | 0.08 | 0 | 0.613 | **SNP10** |
|  |  |  |  |  |  |  |  |  |  | 0.059 | 0.001 | 0.015 | 0 | 0.004 | 0.084 | 0.001 | 0.003 | **SNP11** |
|  |  |  |  |  |  |  |  |  |  |  | 0.02 | 0.237 | 0.002 | 0.04 | 0.723 | 0.008 | 0.041 | **SNP12** |
|  |  |  |  |  |  |  |  |  |  |  |  | 0.071 | 0 | 0.001 | 0.015 | 0 | 0.001 | **SNP13** |
|  |  |  |  |  |  |  |  |  |  |  |  |  | 0 | 0.011 | 0.174 | 0.002 | 0.01 | **SNP14** |
|  |  |  |  |  |  |  |  |  |  |  |  |  |  | 0.01 | 0 | 0 | 0.01 | **SNP15** |
|  |  |  |  |  |  |  |  |  |  |  |  |  |  |  | 0.057 | 0.001 | 1 | **SNP16** |
|  |  |  |  |  |  |  |  |  |  |  |  |  |  |  |  | 0.011 | 0.057 | **SNP17** |
|  |  |  |  |  |  |  |  |  |  |  |  |  |  |  |  |  | 0.001 | **SNP18** |

SNP Legend:

| **SNP1** | rs13266204 | **SNP8** | rs327 | **SNP15** | rs1569209 |
| --- | --- | --- | --- | --- | --- |
| **SNP2** | rs1031045 | **SNP9** | rs328 | **SNP16** | rs1011685 |
| **SNP3** | rs3779788 | **SNP10** | rs12679834 | **SNP17** | rs10096633 |
| **SNP4** | rs1121923 | **SNP11** | rs3289 | **SNP18** | rs11995036 |
| **SNP5** | rs343 | **SNP12** | rs13702 | **SNP19** | rs17482753 |
| **SNP6** | rs258 | **SNP13** | rs9644636 |  |  |
| **SNP7** | rs325 | **SNP14** | rs2197089 |  |  |

b) JHS-EUR-2_LPL_

| **SNP2** | **SNP3** | **SNP4** | **SNP5** | **SNP6** | **SNP7** | **SNP8** | **SNP9** | **SNP10** | **SNP11** | **SNP12** | **SNP13** | **SNP14** | **SNP15** | **SNP16** | **SNP17** | **SNP18** | **SNP19** | **EUR** |
| --- | --- | --- | --- | --- | --- | --- | --- | --- | --- | --- | --- | --- | --- | --- | --- | --- | --- | --- |
| 0.002 | 0.033 | 0 | 0.02 | 0.16 | 0.051 | 0.148 | 0.05 | 0.051 | 0 | 0.159 | 0.003 | 0.017 | 0.036 | 0.051 | 0.065 | 0 | 0.053 | **SNP1** |
|  | 0.001 | 0 | 0.001 | 0.016 | 0.001 | 0.018 | 0.001 | 0.001 | 0 | 0.017 | 0.003 | 0.007 | 0.001 | 0.002 | 0.042 | 0 | 0.002 | **SNP2** |
|  |  | 0.002 | 0.475 | 0.128 | 0.33 | 0.176 | 0.359 | 0.33 | 0 | 0.176 | 0.007 | 0.072 | 0.09 | 0.297 | 0.27 | 0 | 0.303 | **SNP3** |
|  |  |  | 0.001 | 0.033 | 0.002 | 0.008 | 0.002 | 0.002 | 0 | 0.04 | 0.006 | 0.013 | 0.002 | 0.002 | 0.109 | 0 | 0.003 | **SNP4** |
|  |  |  |  | 0.097 | 0.155 | 0.105 | 0.185 | 0.155 | 0 | 0.115 | 0.017 | 0.042 | 0.175 | 0.126 | 0.14 | 0 | 0.143 | **SNP5** |
|  |  |  |  |  | 0.003 | 0.005 | 0 | 0.003 | 0 | 0 | 0.142 | 0.317 | 0 | 0.002 | 0.03 | 0 | 0.001 | **SNP6** |
|  |  |  |  |  |  | 0.358 | 1 | 1 | 0 | 0.309 | 0.055 | 0.118 | 0.704 | 1 | 0.779 | 0 | 1 | **SNP7** |
|  |  |  |  |  |  |  | 0.368 | 0.358 | 0 | 0.928 | 0.153 | 0.345 | 0.252 | 0.351 | 0.335 | 0 | 0.353 | **SNP8** |
|  |  |  |  |  |  |  |  | 1 | 0 | 0.32 | 0.056 | 0.126 | 0.719 | 1 | 0.787 | 0 | 1 | **SNP9** |
|  |  |  |  |  |  |  |  |  | 0 | 0.309 | 0.055 | 0.118 | 0.704 | 1 | 0.779 | 0 | 1 | **SNP10** |
|  |  |  |  |  |  |  |  |  |  | 0 | 0 | 0 | 0 | 0 | 0 | 0 | 0 | **SNP11** |
|  |  |  |  |  |  |  |  |  |  |  | 0.165 | 0.385 | 0.225 | 0.312 | 0.411 | 0 | 0.307 | **SNP12** |
|  |  |  |  |  |  |  |  |  |  |  |  | 0.396 | 0.042 | 0.056 | 0.072 | 0 | 0.059 | **SNP13** |
|  |  |  |  |  |  |  |  |  |  |  |  |  | 0.088 | 0.12 | 0.16 | 0 | 0.12 | **SNP14** |
|  |  |  |  |  |  |  |  |  |  |  |  |  |  | 0.705 | 0.551 | 0 | 0.719 | **SNP15** |
|  |  |  |  |  |  |  |  |  |  |  |  |  |  |  | 0.756 | 0 | 1 | **SNP16** |
|  |  |  |  |  |  |  |  |  |  |  |  |  |  |  |  | 0 | 0.786 | **SNP17** |
|  |  |  |  |  |  |  |  |  |  |  |  |  |  |  |  |  | 0 | **SNP18** |

SNP Legend:

| **SNP1** | rs13266204 | **SNP8** | rs327 | **SNP15** | rs1569209 |
| --- | --- | --- | --- | --- | --- |
| **SNP2** | rs1031045 | **SNP9** | rs328 | **SNP16** | rs1011685 |
| **SNP3** | rs3779788 | **SNP10** | rs12679834 | **SNP17** | rs10096633 |
| **SNP4** | rs1121923 | **SNP11** | rs3289 | **SNP18** | rs11995036 |
| **SNP5** | rs343 | **SNP12** | rs13702 | **SNP19** | rs17482753 |
| **SNP6** | rs258 | **SNP13** | rs9644636 |  |  |
| **SNP7** | rs325 | **SNP14** | rs2197089 |  |  |
