# Supplementary material for: Genetic Differences between the Determinants of Lipid Profile Phenotypes in African and European Americans: The Jackson Heart Study
Source: PLoS Genet. 2009 Jan 16;5(1):e1000342. doi: 10.1371/journal.pgen.1000342 (PMC2613537; doi:10.1371/journal.pgen.1000342)
Supplement: Table S4 — Effect size of LPL variants on TG levels. P-values for association of TRG with genotype in the total population (p_trg_all) and JHS-AFR-2LPL (p_trg_afr); SNPs with p<0.0006 are shown in bold. The p-value for interaction is for the significance of the genotype×local ancestry term in the linear regression model for the total population (p<0.05 is shown in bold). The percent change in TG level per LPL allele is shown with standard error (SE) for the total population (effect_all), JHS-AFR-2LPL (effect_afr) and JHS-EUR-1_2LPL (effect_eur). The SNP frequencies in the total population (f_all), JHS-AFR-2LPL (f_afr) and JHS-EUR-2LPL (f_eur) are also shown, as well as the chromosomal position in bases on chromosome 8. (0.19 MB DOC) [file pgen.1000342.s005.doc]

**Supplementary Table 4: Effect size of *LPL* variants on TG levels.** P-values for association of TRG with genotype in the total population (p_trg_all) and JHS-AFR-2*_LPL_* (p_trg_afr); SNPs with p<0.0006 are shown in bold. The p-value for interaction is for the significance of the genotype x local ancestry term in the linear regression model for the total population (p<0.05 is shown in bold). The percent change in TG level per *LPL* allele is shown with standard error (SE) for the total population (effect_all), JHS-AFR-2*_LPL_* (effect_afr) and JHS-EUR-1_2*_LPL_* (effect_eur). The SNP frequencies in the total population (f_all), JHS-AFR-2*_LPL_* (f_afr) and JHS-EUR-2*_LPL_* (f_eur) are also shown, as well as the chromosomal position in bases on chromosome 8.

| **SNP** | **Position** | **f_afr** | **f_eur** | **f_all** | **p_trg_all** | **p_int_trg** | **p_trg_afr** | **effect_all** | **SE** | **effect_afr** | **SE** | **effect_eur** | **SE** |
| --- | --- | --- | --- | --- | --- | --- | --- | --- | --- | --- | --- | --- | --- |
| rs7816032 | 19831171 | 0.25 | 0.84 | 0.34 | 0.71 | 0.16 | 0.51 | 0.50 | 1.40 | 1.2 | 1.9 | 1.7 | 3.1 |
| rs7009128 | 19831808 | 0.65 | 0.99 | 0.71 | 0.057 | 0.82 | 0.50 | 2.7 | 1.4 | 1.2 | 1.7 | 4.0 | 4.0 |
| rs1470186 | 19840069 | 0.78 | 0.99 | 0.82 | 0.025 | 0.52 | 0.11 | 3.7 | 1.6 | 3.1 | 1.9 | 4.7 | 4.7 |
| rs17091742 | 19840410 | 0.78 | 0.99 | 0.82 | 0.062 | 0.84 | 0.36 | 3.2 | 1.6 | 1.8 | 2.0 | 6.1 | 4.7 |
| rs1800590 | 19840951 | 0.53 | 0.98 | 0.62 | 0.26 | 0.55 | 0.81 | 1.6 | 1.3 | 0.09 | 1.64 | 4.0 | 3.9 |
| rs17410577 | 19843825 | 0.89 | 0.79 | 0.87 | 0.64 | 0.38 | 0.83 | 0.90 | 1.85 | 0.31 | 2.58 | 5.2 | 3.7 |
| rs1534649 | 19843921 | 0.07 | 0.59 | 0.16 | 0.82 | 0.83 | 0.62 | 0.22 | 1.95 | 1.6 | 3.2 | 0.28 | 3.33 |
| rs13266204 | 19844285 | 0.95 | 0.76 | 0.92 | 0.68 | 0.13 | 0.27 | 0.92 | 2.23 | 3.8 | 3.6 | 4.5 | 3.8 |
| rs1031045 | 19845392 | 0.54 | 0.99 | 0.63 | 0.025 | 0.85 | 0.22 | 3.1 | 1.4 | 2.1 | 1.6 | 2.6 | 3.9 |
| rs3779788 | 19847373 | 0.98 | 0.91 | 0.95 | 1.22E-03 | 0.60 | 0.14 | 10.4 | 3.0 | 8.6 | 5.4 | 9.3 | 4.5 |
| rs7000460 | 19848082 | 0.74 | 1.00 | 0.78 | 5.53E-03 | 0.69 | 0.051 | 4.4 | 1.5 | 3.7 | 1.8 | 0.12 | 4.39 |
| rs11542065 | 19850095 | 0.98 | 1.00 | 0.99 | 0.85 | 0.11 | 0.44 | 0.35 | 5.63 | 5.3 | 6.7 | 15.4 | 17.6 |
| rs6991305 | 19850661 | 0.96 | 1.00 | 0.97 | 0.38 | 0.25 | 0.50 | 2.9 | 3.3 | 3.1 | 4.1 | 14.8 | 9.3 |
| rs7002728 | 19853017 | 0.84 | 0.99 | 0.87 | 0.26 | 0.18 | 0.18 | 2.2 | 1.9 | 3.1 | 2.3 | 4.9 | 5.5 |
| rs1121923 | 19853715 | 0.87 | 0.98 | 0.88 | 0.38 | 0.44 | 0.39 | 1.7 | 2.0 | 1.9 | 2.4 | 8.5 | 5.1 |
| rs343 | 19855067 | 0.95 | 0.94 | 0.95 | 1.34E-03 | 0.27 | 0.16 | 9.2 | 2.7 | 5.7 | 3.8 | 12.9 | 5.3 |
| rs248 | 19855106 | 0.96 | 0.96 | 0.96 | 0.82 | 0.20 | 0.69 | 0.25 | 3.09 | 1.4 | 4.3 | 3.1 | 6.4 |
| rs249 | 19855286 | 0.85 | 0.93 | 0.87 | 0.059 | 0.13 | 0.11 | 3.6 | 1.8 | 3.9 | 2.3 | 3.5 | 4.4 |
| rs251 | 19855440 | 0.85 | 1.00 | 0.87 | 0.85 | 0.72 | 0.63 | 0.12 | 1.82 | 1.1 | 2.3 | 3.4 | 4.6 |
| rs253 | 19855697 | 0.21 | 0.59 | 0.27 | 0.72 | 0.10 | 0.28 | 0.47 | 1.46 | 2.2 | 2.0 | 0.42 | 2.85 |
| rs255 | 19856181 | 0.66 | 0.76 | 0.66 | 0.078 | 0.13 | 0.16 | 2.7 | 1.5 | 2.9 | 1.9 | 6.5 | 3.4 |
| rs258 | 19856532 | 0.04 | 0.60 | 0.13 | 0.11 | 0.84 | 0.075 | 3.6 | 2.2 | 7.8 | 4.2 | 0.61 | 3.31 |
| rs260 | 19856789 | 0.91 | 1.00 | 0.93 | 0.87 | 0.11 | 0.86 | 0.04 | 2.36 | 0.15 | 2.89 | 12.1 | 6.9 |
| rs261 | 19856900 | 0.83 | 1.00 | 0.85 | 0.096 | 0.06 | 0.13 | 3.1 | 1.8 | 3.3 | 2.2 | 4.8 | 5.2 |
| rs263 | 19857092 | 0.61 | 0.86 | 0.64 | 8.31E-03 | 0.092 | 0.12 | 3.5 | 1.3 | 2.6 | 1.7 | 5.6 | 3.1 |
| rs264 | 19857460 | 0.87 | 0.87 | 0.86 | 5.28E-03 | 0.23 | 0.089 | 5.1 | 1.7 | 4.2 | 2.3 | 9.2 | 3.9 |
| rs266 | 19857579 | 0.86 | 1.00 | 0.88 | 0.75 | 0.15 | 0.84 | 0.58 | 1.91 | 0.27 | 2.36 | 7.8 | 5.3 |
| rs268 | 19857809 | 1.00 | 0.98 | 1.00 | 0.77 | 0.36 | 0.10 | 1.6 | 10.6 | 45.2 | 26.6 | 4.4 | 12.8 |
| rs270 | 19857956 | 0.92 | 0.84 | 0.90 | 0.23 | 0.32 | 0.28 | 2.6 | 2.0 | 3.3 | 3.0 | 3.6 | 4.0 |
| rs279 | 19858976 | 0.84 | 0.99 | 0.87 | 0.47 | 0.11 | 0.18 | 1.4 | 1.9 | 3.2 | 2.3 | 3.8 | 5.9 |
| rs280 | 19859162 | 0.94 | 0.99 | 0.95 | 0.026 | 0.56 | 0.013 | 7.0 | 3.0 | 9.6 | 3.6 | 2.8 | 8.4 |
| rs17091775 | 19859268 | 0.94 | 0.99 | 0.95 | 0.052 | 0.35 | 0.010 | 5.9 | 2.9 | 9.7 | 3.5 | 0.57 | 8.49 |
| rs281 | 19859303 | 0.56 | 0.77 | 0.59 | 0.21 | 0.16 | 0.16 | 1.6 | 1.3 | 2.4 | 1.7 | 0.91 | 2.77 |
| rs292 | 19860335 | 0.99 | 1.00 | 0.99 | 0.11 | 0.74 | 0.22 | 9.2 | 5.6 | 9.1 | 7.2 | 10.6 | 12.9 |
| rs295 | 19860518 | 0.57 | 0.73 | 0.61 | 3.40E-03 | 0.61 | 0.0088 | 3.9 | 1.3 | 4.5 | 1.6 | 1.6 | 2.9 |
| rs297 | 19860651 | 0.66 | 0.74 | 0.68 | 0.038 | 0.86 | 0.19 | 2.9 | 1.3 | 2.3 | 1.7 | 1.8 | 3.1 |
| rs301 | 19861214 | 0.66 | 0.74 | 0.68 | 0.041 | 0.85 | 0.21 | 2.8 | 1.3 | 2.2 | 1.7 | 2.0 | 3.0 |
| rs312 | 19862277 | 0.71 | 0.90 | 0.75 | 0.84 | 0.09 | 0.45 | 0.11 | 1.42 | 1.4 | 1.8 | 5.8 | 3.5 |
| rs316 | 19862716 | 0.74 | 0.89 | 0.77 | 0.46 | 0.081 | 0.37 | 1.1 | 1.4 | 1.6 | 1.9 | 4.8 | 3.5 |
| rs5934 | 19862831 | 0.97 | 1.00 | 0.97 | 0.42 | 0.83 | 0.54 | 2.9 | 3.5 | 2.5 | 4.4 | 5.6 | 9.6 |
| rs325 | 19863608 | 0.94 | 0.86 | 0.93 | **4.5E-05** | **0.033** | 0.23 | 10.3 | 2.4 | 4.3 | 3.4 | 19.3 | 4.4 |
| rs327 | 19863816 | 0.55 | 0.68 | 0.59 | 7.82E-04 | 0.86 | 0.0083 | 4.3 | 1.2 | 4.4 | 1.6 | 3.7 | 2.8 |
| rs328 | 19864004 | 0.94 | 0.86 | 0.93 | **7.1E-05** | 0.052 | 0.22 | 10.2 | 2.4 | 4.5 | 3.4 | 18.6 | 4.5 |
| rs330 | 19864676 | 0.91 | 0.83 | 0.90 | 0.78 | 0.23 | 0.81 | 0.50 | 1.95 | 0.30 | 2.71 | 2.7 | 4.0 |
| rs331 | 19864685 | 0.56 | 0.68 | 0.60 | 0.012 | 0.72 | 0.029 | 3.2 | 1.2 | 3.6 | 1.6 | 1.7 | 2.8 |
| rs12679834 | 19864713 | 0.92 | 0.86 | 0.91 | **2.71E-04** | **0.013** | 0.33 | 8.3 | 2.1 | 3.1 | 3.0 | 17.8 | 4.3 |
| rs17116619 | 19865513 | 0.90 | 1.00 | 0.92 | 0.100 | 0.56 | 0.021 | 4.0 | 2.3 | 6.7 | 2.7 | 1.3 | 7.0 |
| rs10283151 | 19866974 | 0.83 | 0.99 | 0.86 | 0.38 | 0.10 | 0.15 | 1.7 | 1.9 | 3.4 | 2.3 | 6.7 | 5.5 |
| rs4922115 | 19867110 | 0.90 | 0.85 | 0.89 | 0.35 | 0.44 | 0.48 | 1.9 | 1.9 | 2.1 | 2.6 | 3.9 | 4.1 |
| rs7818177 | 19867130 | 0.98 | 1.00 | 0.99 | 0.056 | 0.048 | 0.035 | 9.9 | 5.0 | 13.5 | 6.1 | 5.7 | 16.4 |
| rs3289 | 19867472 | 0.92 | 1.00 | 0.92 | 8.13E-03 | 0.23 | 0.22 | 7.0 | 2.5 | 3.8 | 3.0 | 10.3 | 7.5 |
| rs11570892 | 19867897 | 0.75 | 0.82 | 0.77 | 0.42 | 0.41 | 0.33 | 1.1 | 1.4 | 1.8 | 1.8 | 1.1 | 3.1 |
| rs13702 | 19868772 | 0.43 | 0.66 | 0.48 | 1.10E-03 | 0.81 | 0.042 | 4.1 | 1.2 | 3.4 | 1.6 | 2.2 | 2.8 |
| rs1059611 | 19868843 | 0.79 | 0.83 | 0.81 | 2.05E-03 | 0.23 | 0.11 | 4.9 | 1.5 | 3.3 | 1.9 | 8.2 | 3.5 |
| rs17091815 | 19868884 | 0.92 | 1.00 | 0.93 | 0.14 | 0.81 | 0.071 | 3.9 | 2.5 | 5.8 | 3.0 | 6.3 | 7.8 |
| rs15285 | 19868947 | 0.43 | 0.67 | 0.48 | 5.89E-03 | 0.70 | 0.047 | 3.5 | 1.2 | 3.3 | 1.6 | 0.30 | 2.72 |
| rs3916027 | 19869148 | 0.55 | 0.69 | 0.58 | 4.61E-03 | 0.77 | 0.025 | 3.6 | 1.2 | 3.8 | 1.6 | 2.2 | 2.8 |
| rs9644636 | 19869176 | 0.99 | 0.75 | 0.94 | 0.78 | 0.51 | 0.72 | 0.65 | 2.69 | 2.5 | 6.7 | 1.2 | 3.6 |
| rs4921684 | 19869408 | 0.93 | 0.86 | 0.92 | 0.78 | 0.26 | 0.80 | 0.59 | 2.20 | 0.62 | 3.16 | 3.0 | 4.3 |
| rs2197089 | 19870653 | 0.15 | 0.41 | 0.21 | 9.30E-03 | 0.72 | 0.027 | 4.1 | 1.5 | 5.1 | 2.2 | 1.3 | 2.8 |
| rs10105606 | 19872128 | 0.26 | 0.66 | 0.34 | 0.032 | 0.74 | 0.14 | 2.9 | 1.3 | 2.7 | 1.8 | 2.2 | 2.8 |
| rs2898493 | 19873001 | 0.76 | 0.99 | 0.80 | 0.27 | 0.18 | 0.099 | 1.8 | 1.6 | 3.4 | 1.9 | 7.9 | 4.6 |
| rs2410617 | 19873173 | 0.90 | 0.85 | 0.89 | 0.23 | 0.32 | 0.53 | 2.4 | 1.9 | 1.8 | 2.6 | 3.7 | 4.0 |
| rs7845291 | 19873983 | 0.69 | 0.98 | 0.75 | 0.057 | 0.37 | 0.025 | 2.9 | 1.4 | 4.2 | 1.7 | 4.4 | 4.2 |
| rs1569209 | 19874450 | 1.00 | 0.90 | 0.98 | 9.86E-04 | 0.86 | 0.62 | 16.6 | 4.7 | 17.3 | 32.5 | 18.9 | 5.6 |
| rs6651471 | 19874626 | 0.90 | 0.99 | 0.92 | 0.80 | 0.21 | 0.44 | 0.39 | 2.32 | 2.1 | 2.8 | 4.5 | 7.2 |
| rs6651484 | 19874789 | 0.70 | 0.98 | 0.76 | 0.070 | 0.27 | 0.022 | 2.8 | 1.5 | 4.4 | 1.8 | 5.9 | 4.3 |
| rs1561750 | 19875046 | 0.58 | 0.83 | 0.63 | 0.31 | 0.03 | 0.037 | 1.3 | 1.3 | 3.6 | 1.6 | 5.3 | 3.1 |
| rs1011685 | 19875049 | 0.95 | 0.87 | 0.93 | **2.0E-05** | **0.027** | 0.25 | 11.5 | 2.5 | 4.4 | 3.6 | 21.5 | 4.5 |
| rs10095784 | 19875162 | 0.86 | 0.99 | 0.88 | 0.37 | 0.15 | 0.21 | 1.8 | 1.9 | 3.1 | 2.3 | 5.3 | 5.9 |
| rs10096633 | 19875201 | 0.50 | 0.82 | 0.57 | **2.7E-06** | 0.15 | 0.0071 | 6.2 | 1.2 | 4.5 | 1.6 | 6.6 | 3.1 |
| rs11995036 | 19875365 | 0.99 | 1.00 | 0.99 | 0.050 | 0.84 | 0.018 | 12.9 | 6.1 | 21.3 | 7.9 | 18.4 | 23.3 |
| rs17091870 | 19875910 | 0.92 | 0.99 | 0.93 | 0.085 | 0.32 | 0.035 | 4.4 | 2.5 | 6.7 | 3.0 | 9.2 | 6.8 |
| rs1372339 | 19876078 | 0.84 | 0.85 | 0.85 | 0.075 | 0.56 | 0.48 | 3.1 | 1.7 | 1.7 | 2.2 | 2.7 | 3.8 |
| rs17091872 | 19876257 | 0.81 | 0.84 | 0.81 | 5.72E-03 | 0.27 | 0.25 | 4.4 | 1.5 | 2.4 | 2.0 | 4.6 | 3.5 |
| rs17482753 | 19876926 | 0.95 | 0.85 | 0.94 | **4.9E-05** | **0.023** | 0.33 | 10.8 | 2.5 | 3.7 | 3.6 | 21.4 | 4.5 |
| rs4922116 | 19877058 | 0.85 | 0.85 | 0.85 | 0.072 | 0.49 | 0.42 | 3.4 | 1.8 | 2.0 | 2.3 | 3.3 | 4.1 |
| rs7014261 | 19877672 | 0.93 | 1.00 | 0.94 | 0.80 | 0.25 | 0.68 | 0.45 | 2.66 | 1.1 | 3.2 | 13.0 | 7.7 |
| rs1441777 | 19877851 | 0.88 | 0.84 | 0.88 | 0.39 | 0.38 | 0.64 | 1.6 | 1.8 | 1.3 | 2.4 | 3.7 | 3.5 |
| rs10097668 | 19878009 | 0.91 | 0.77 | 0.88 | 0.74 | 0.57 | 0.84 | 0.59 | 1.87 | 0.05 | 2.81 | 1.6 | 3.5 |
| rs11988512 | 19879436 | 0.77 | 0.97 | 0.81 | 0.070 | 0.59 | 0.11 | 3.0 | 1.6 | 3.3 | 2.0 | 5.6 | 4.2 |
| rs10105868 | 19880231 | 0.96 | 1.00 | 0.97 | 0.73 | 0.75 | 0.57 | 1.1 | 3.3 | 2.6 | 4.1 | 8.8 | 10.4 |
| rs6586883 | 19882749 | 0.25 | 0.68 | 0.34 | 0.043 | 0.79 | 0.033 | 2.8 | 1.3 | 4.1 | 1.8 | 1.1 | 2.8 |
| rs7843168 | 19885738 | 0.83 | 0.98 | 0.86 | 0.24 | 0.58 | 0.57 | 2.2 | 1.8 | 1.2 | 2.2 | 8.9 | 4.7 |
| rs7825274 | 19886468 | 0.87 | 0.98 | 0.89 | 0.25 | 0.84 | 0.74 | 2.3 | 1.9 | 0.65 | 2.35 | 3.8 | 5.2 |
