# Supplementary material for: Genetic Differences between the Determinants of Lipid Profile Phenotypes in African and European Americans: The Jackson Heart Study
Source: PLoS Genet. 2009 Jan 16;5(1):e1000342. doi: 10.1371/journal.pgen.1000342 (PMC2613537; doi:10.1371/journal.pgen.1000342)
Supplement: Table S5 — Effect size of LPL variants on HDL-C levels. P-values for association of HDL-C with genotype in the total population (p_hdl_all) and for the significance of the genotype×local ancestry term in the linear regression model. SNPs with p<0.0006 are shown in bold. The change in HDL-C level (mg/dL) per LPL allele is shown with standard error for the total population (effect_all), JHS-AFR-2LPL (effect_afr) and JHS-EUR-1_2LPL (effect_eur). The SNP frequencies in the total population (f_all), JHS-AFR-2LPL (f_afr) and JHS-EUR-2LPL (f_eur) are also shown, as well as the chromosomal position in bases on chromosome 8. (0.18 MB DOC) [file pgen.1000342.s006.doc]

**Supplementary Table 5: Effect size of *LPL* variants on HDL-C levels.** P-values for association of HDL-C with genotype in the total population (p_hdl_all) and for the significance of the genotype x local ancestry term in the linear regression model. SNPs with p<0.0006 are shown in bold. The change in HDL-C level (mg/dL) per *LPL* allele is shown with standard error for the total population (effect_all), JHS-AFR-2*_LPL_* (effect_afr) and JHS-EUR-1_2*_LPL_* (effect_eur). The SNP frequencies in the total population (f_all), JHS-AFR-2*_LPL_* (f_afr) and JHS-EUR-2*_LPL_* (f_eur) are also shown, as well as the chromosomal position in bases on chromosome 8.

| **SNP** | **Position** | **f_afr** | **f_eur** | **f_all** | **p_hdl_all** | **p_int_hdl** | **effect_all** | **SE** | **effect_afr** | **SE** | **effect_eur** | **SE** |
| --- | --- | --- | --- | --- | --- | --- | --- | --- | --- | --- | --- | --- |
| rs10096633 | 19875201 | 0.50 | 0.82 | 0.57 | 1.42E-03 | 0.71 | 1.10 | 0.34 | 1.20 | 0.44 | 0.60 | 0.76 |
| rs1011685 | 19875049 | 0.95 | 0.87 | 0.93 | 2.37E-03 | 0.34 | 2.07 | 0.66 | 1.43 | 0.98 | 1.68 | 1.10 |
| rs325 | 19863608 | 0.94 | 0.86 | 0.93 | 9.52E-04 | 0.17 | -2.15 | 0.63 | -1.38 | 0.93 | -2.13 | 1.10 |
| rs17482753 | 19876926 | 0.95 | 0.85 | 0.94 | 1.45E-03 | 0.23 | 2.19 | 0.67 | 1.45 | 0.99 | 1.97 | 1.12 |
| rs328 | 19864004 | 0.94 | 0.86 | 0.93 | 6.69E-04 | 0.23 | 2.24 | 0.64 | 1.57 | 0.95 | 1.79 | 1.10 |
| rs12679834 | 19864713 | 0.92 | 0.86 | 0.91 | 3.72E-03 | 0.21 | -1.73 | 0.58 | -1.12 | 0.83 | -1.63 | 1.07 |
| rs327 | 19863816 | 0.55 | 0.68 | 0.59 | 1.56E-03 | 0.52 | -1.08 | 0.33 | -1.30 | 0.45 | -1.05 | 0.71 |
| rs1569209 | 19874450 | 1.00 | 0.90 | 0.98 | 7.69E-03 | 0.78 | -3.38 | 1.24 | -7.60 | 8.22 | -2.80 | 1.38 |
| rs13702 | 19868772 | 0.43 | 0.66 | 0.48 | **1.21E-04** | 0.73 | -1.28 | 0.33 | -1.69 | 0.44 | -0.59 | 0.69 |
| rs3779788 | 19847373 | 0.98 | 0.91 | 0.95 | 0.057 | 0.85 | 1.56 | 0.80 | 1.85 | 1.49 | 0.50 | 1.12 |
| rs343 | 19855067 | 0.95 | 0.94 | 0.95 | 1.18E-03 | 0.85 | -2.43 | 0.72 | -2.80 | 1.05 | -1.77 | 1.31 |
| rs1059611 | 19868843 | 0.79 | 0.83 | 0.81 | 0.018 | 0.85 | -0.99 | 0.40 | -1.23 | 0.53 | -0.07 | 0.88 |
| rs295 | 19860518 | 0.57 | 0.73 | 0.61 | 8.28E-03 | 0.64 | 0.92 | 0.34 | 1.19 | 0.46 | 0.92 | 0.72 |
| rs3916027 | 19869148 | 0.55 | 0.69 | 0.58 | 3.43E-03 | 0.41 | -0.99 | 0.33 | -1.11 | 0.45 | -1.20 | 0.68 |
| rs264 | 19857460 | 0.87 | 0.87 | 0.86 | 0.093 | 0.83 | -0.81 | 0.47 | -1.16 | 0.64 | 0.06 | 0.97 |
| rs7000460 | 19848082 | 0.74 | 1.00 | 0.78 | 0.046 | 0.80 | -0.84 | 0.41 | -0.95 | 0.52 | 0.05 | 1.08 |
| rs17091872 | 19876257 | 0.81 | 0.84 | 0.81 | 0.64 | 0.74 | 0.19 | 0.41 | -0.18 | 0.55 | 0.02 | 0.88 |
| rs15285 | 19868947 | 0.43 | 0.67 | 0.48 | 6.91E-04 | 0.44 | 1.12 | 0.32 | 1.62 | 0.44 | 0.16 | 0.67 |
| rs3289 | 19867472 | 0.92 | 1.00 | 0.92 | 1.98E-03 | 0.58 | 2.12 | 0.67 | 2.64 | 0.83 | 1.92 | 1.79 |
| rs263 | 19857092 | 0.61 | 0.86 | 0.64 | 0.012 | 0.86 | 0.90 | 0.35 | 1.05 | 0.46 | 0.29 | 0.77 |
| rs2197089 | 19870653 | 0.15 | 0.41 | 0.21 | 2.14E-03 | 0.84 | -1.29 | 0.41 | -1.99 | 0.62 | -0.73 | 0.71 |
| rs331 | 19864685 | 0.56 | 0.68 | 0.60 | 6.10E-03 | 0.69 | -0.92 | 0.32 | -1.13 | 0.44 | -0.71 | 0.70 |
| rs1470186 | 19840069 | 0.78 | 0.99 | 0.82 | 0.074 | 0.84 | 0.79 | 0.43 | 0.78 | 0.54 | 1.18 | 1.16 |
| rs1031045 | 19845392 | 0.54 | 0.99 | 0.63 | 0.45 | 0.75 | 0.28 | 0.36 | 0.09 | 0.46 | 0.37 | 0.95 |
| rs280 | 19859162 | 0.94 | 0.99 | 0.95 | 0.72 | 0.60 | -0.27 | 0.79 | -0.07 | 0.99 | -1.77 | 1.99 |
| rs10105606 | 19872128 | 0.26 | 0.66 | 0.34 | 4.47E-03 | 0.82 | -1.03 | 0.36 | -1.48 | 0.50 | -0.66 | 0.70 |
| rs297 | 19860651 | 0.66 | 0.74 | 0.68 | 0.016 | 0.65 | -0.89 | 0.35 | -1.06 | 0.48 | -0.56 | 0.76 |
| rs301 | 19861214 | 0.66 | 0.74 | 0.68 | 8.79E-03 | 0.82 | -0.94 | 0.35 | -1.20 | 0.47 | -0.61 | 0.73 |
| rs6586883 | 19882749 | 0.25 | 0.68 | 0.34 | 0.019 | 0.77 | 0.86 | 0.36 | 1.36 | 0.51 | 0.18 | 0.70 |
| rs11995036 | 19875365 | 0.99 | 1.00 | 0.99 | 0.24 | 0.75 | -1.97 | 1.59 | -2.97 | 2.12 | -7.04 | 4.87 |
| rs17091775 | 19859268 | 0.94 | 0.99 | 0.95 | 0.74 | 0.61 | -0.25 | 0.77 | -0.19 | 0.96 | -1.95 | 2.01 |
| rs7818177 | 19867130 | 0.98 | 1.00 | 0.99 | 0.87 | 0.25 | -0.04 | 1.32 | 0.68 | 1.68 | -2.61 | 3.68 |
| rs7845291 | 19873983 | 0.69 | 0.98 | 0.75 | 0.41 | 0.56 | -0.33 | 0.39 | -0.66 | 0.49 | -0.16 | 1.05 |
| rs7009128 | 19831808 | 0.65 | 0.99 | 0.71 | 0.14 | 0.47 | 0.56 | 0.37 | 0.69 | 0.46 | -0.10 | 0.98 |
| rs249 | 19855286 | 0.85 | 0.93 | 0.87 | 0.020 | 0.64 | 1.18 | 0.49 | 1.08 | 0.64 | 1.69 | 1.08 |
| rs17091742 | 19840410 | 0.78 | 0.99 | 0.82 | 0.22 | 0.78 | -0.56 | 0.44 | -0.56 | 0.56 | -0.29 | 1.16 |
| rs6651484 | 19874789 | 0.70 | 0.98 | 0.76 | 0.37 | 0.72 | -0.36 | 0.40 | -0.66 | 0.50 | -0.46 | 1.06 |
| rs11988512 | 19879436 | 0.77 | 0.97 | 0.81 | 0.38 | 0.61 | 0.39 | 0.43 | 0.52 | 0.55 | -0.73 | 1.05 |
| rs4922116 | 19877058 | 0.85 | 0.85 | 0.85 | 0.32 | 0.46 | -0.51 | 0.49 | -0.99 | 0.66 | -0.34 | 1.05 |
| rs1372339 | 19876078 | 0.84 | 0.85 | 0.85 | 0.35 | 0.72 | 0.44 | 0.45 | 0.81 | 0.62 | 0.60 | 0.94 |
| rs255 | 19856181 | 0.66 | 0.76 | 0.66 | 0.031 | 0.52 | -0.90 | 0.40 | -1.04 | 0.54 | -1.27 | 0.84 |
| rs17091870 | 19875910 | 0.92 | 0.99 | 0.93 | 0.85 | 0.26 | 0.05 | 0.66 | -0.23 | 0.83 | 1.10 | 1.65 |
| rs261 | 19856900 | 0.83 | 1.00 | 0.85 | 0.027 | 0.11 | -1.09 | 0.48 | -1.69 | 0.61 | -0.54 | 1.26 |
| rs17116619 | 19865513 | 0.90 | 1.00 | 0.92 | 0.67 | 0.30 | 0.27 | 0.62 | 0.14 | 0.76 | 2.75 | 1.69 |
| rs258 | 19856532 | 0.04 | 0.60 | 0.13 | 0.37 | 0.85 | 0.55 | 0.59 | 0.58 | 1.15 | 1.00 | 0.83 |
| rs292 | 19860335 | 0.99 | 1.00 | 0.99 | 0.63 | 0.77 | 0.74 | 1.47 | 1.06 | 1.96 | 2.29 | 3.09 |
| rs17091815 | 19868884 | 0.92 | 1.00 | 0.93 | 0.39 | 0.27 | 0.60 | 0.68 | 0.43 | 0.84 | 4.34 | 1.86 |
| rs281 | 19859303 | 0.56 | 0.77 | 0.59 | 0.51 | 0.69 | 0.23 | 0.34 | 0.09 | 0.46 | 0.64 | 0.70 |
| rs270 | 19857956 | 0.92 | 0.84 | 0.90 | 0.56 | 0.82 | 0.33 | 0.55 | 0.39 | 0.83 | 0.77 | 1.01 |
| rs2410617 | 19873173 | 0.90 | 0.85 | 0.89 | 0.083 | 0.83 | -0.92 | 0.51 | -1.18 | 0.73 | -1.62 | 0.98 |
| rs7843168 | 19885738 | 0.83 | 0.98 | 0.86 | 0.24 | 0.13 | -0.57 | 0.48 | -0.83 | 0.61 | 2.36 | 1.16 |
| rs7825274 | 19886468 | 0.87 | 0.98 | 0.89 | 0.24 | 0.03 | -0.62 | 0.51 | -1.09 | 0.65 | 3.19 | 1.28 |
| rs1800590 | 19840951 | 0.53 | 0.98 | 0.62 | 0.84 | 0.62 | -0.06 | 0.36 | -0.24 | 0.46 | 0.38 | 0.96 |
| rs7002728 | 19853017 | 0.84 | 0.99 | 0.87 | 0.11 | 0.38 | 0.80 | 0.49 | 1.25 | 0.62 | -0.78 | 1.37 |
| rs2898493 | 19873001 | 0.76 | 0.99 | 0.80 | 0.50 | 0.12 | -0.30 | 0.42 | -0.95 | 0.53 | 1.02 | 1.12 |
| rs1561750 | 19875046 | 0.58 | 0.83 | 0.63 | 0.15 | 0.80 | -0.50 | 0.34 | -0.69 | 0.45 | -1.06 | 0.76 |
| rs4922115 | 19867110 | 0.90 | 0.85 | 0.89 | 0.25 | 0.83 | -0.62 | 0.52 | -0.74 | 0.73 | -0.86 | 1.01 |
| rs10095784 | 19875162 | 0.86 | 0.99 | 0.88 | 0.37 | 0.02 | -0.47 | 0.51 | -1.35 | 0.64 | 2.08 | 1.45 |
| rs10283151 | 19866974 | 0.83 | 0.99 | 0.86 | 0.56 | 0.09 | -0.30 | 0.50 | -1.01 | 0.62 | 1.69 | 1.37 |
| rs6991305 | 19850661 | 0.96 | 1.00 | 0.97 | 0.87 | 0.41 | 0.07 | 0.88 | 0.18 | 1.12 | -4.69 | 2.29 |
| rs1121923 | 19853715 | 0.87 | 0.98 | 0.88 | 0.12 | 0.72 | -0.85 | 0.53 | -0.75 | 0.68 | 0.02 | 1.31 |
| rs1441777 | 19877851 | 0.88 | 0.84 | 0.88 | 0.26 | 0.58 | -0.55 | 0.47 | -0.24 | 0.68 | -1.03 | 0.89 |
| rs5934 | 19862831 | 0.97 | 1.00 | 0.97 | 0.77 | 0.86 | 0.22 | 0.93 | 0.24 | 1.20 | -1.84 | 2.32 |
| rs11570892 | 19867897 | 0.75 | 0.82 | 0.77 | 0.13 | 0.19 | 0.58 | 0.37 | 0.29 | 0.51 | 1.68 | 0.78 |
| rs316 | 19862716 | 0.74 | 0.89 | 0.77 | 0.20 | 0.15 | -0.51 | 0.39 | -1.11 | 0.51 | 0.32 | 0.89 |
| rs279 | 19858976 | 0.84 | 0.99 | 0.87 | 0.82 | 0.12 | 0.10 | 0.51 | 0.74 | 0.64 | -1.04 | 1.47 |
| rs17410577 | 19843825 | 0.89 | 0.79 | 0.87 | 0.093 | 0.23 | 0.85 | 0.50 | 0.56 | 0.71 | 1.77 | 0.91 |
| rs13266204 | 19844285 | 0.95 | 0.76 | 0.92 | 0.070 | 0.07 | -1.12 | 0.60 | 0.13 | 0.99 | -1.93 | 0.94 |
| rs7816032 | 19831171 | 0.25 | 0.84 | 0.34 | 0.73 | 0.14 | -0.13 | 0.38 | -0.63 | 0.52 | 0.51 | 0.77 |
| rs253 | 19855697 | 0.21 | 0.59 | 0.27 | 0.18 | 0.83 | -0.54 | 0.39 | -0.62 | 0.56 | -1.19 | 0.70 |
| rs10105868 | 19880231 | 0.96 | 1.00 | 0.97 | 0.79 | 0.26 | -0.21 | 0.89 | 0.33 | 1.14 | -0.88 | 2.50 |
| rs10097668 | 19878009 | 0.91 | 0.77 | 0.88 | 0.71 | 0.82 | -0.19 | 0.50 | -0.35 | 0.79 | -0.16 | 0.88 |
| rs266 | 19857579 | 0.86 | 1.00 | 0.88 | 0.85 | 0.33 | -0.07 | 0.51 | -0.17 | 0.65 | 1.21 | 1.30 |
| rs268 | 19857809 | 1.00 | 0.98 | 1.00 | 0.65 | 0.46 | 1.28 | 2.80 | -0.18 | 6.73 | 3.32 | 3.16 |
| rs9644636 | 19869176 | 0.99 | 0.75 | 0.94 | 0.42 | 0.26 | -0.60 | 0.72 | -0.58 | 1.81 | -0.92 | 0.90 |
| rs4921684 | 19869408 | 0.93 | 0.86 | 0.92 | 0.20 | 0.81 | 0.79 | 0.59 | 0.88 | 0.88 | 0.73 | 1.07 |
| rs330 | 19864676 | 0.91 | 0.83 | 0.90 | 0.11 | 0.68 | -0.87 | 0.52 | -1.35 | 0.76 | -0.77 | 0.99 |
| rs6651471 | 19874626 | 0.90 | 0.99 | 0.92 | 0.28 | 0.07 | -0.70 | 0.62 | 0.03 | 0.77 | -3.91 | 1.78 |
| rs7014261 | 19877672 | 0.93 | 1.00 | 0.94 | 0.30 | 0.52 | 0.75 | 0.71 | 1.45 | 0.90 | 1.79 | 1.84 |
| rs248 | 19855106 | 0.96 | 0.96 | 0.96 | 0.42 | 0.64 | -0.70 | 0.82 | -1.12 | 1.19 | -0.35 | 1.59 |
| rs1534649 | 19843921 | 0.07 | 0.59 | 0.16 | 0.31 | 0.85 | -0.55 | 0.52 | -0.79 | 0.88 | -0.93 | 0.83 |
| rs312 | 19862277 | 0.71 | 0.90 | 0.75 | 0.25 | 0.13 | -0.45 | 0.38 | -1.16 | 0.50 | 0.34 | 0.86 |
| rs251 | 19855440 | 0.85 | 1.00 | 0.87 | 0.84 | 0.82 | 0.05 | 0.49 | 0.02 | 0.63 | 0.05 | 1.13 |
| rs11542065 | 19850095 | 0.98 | 1.00 | 0.99 | 0.86 | 0.37 | 0.11 | 1.47 | 0.39 | 1.81 | -1.98 | 4.16 |
| rs260 | 19856789 | 0.91 | 1.00 | 0.93 | 0.86 | 0.23 | -0.05 | 0.64 | -0.37 | 0.82 | 1.25 | 1.68 |
