# Supplementary material for: Genetic Differences between the Determinants of Lipid Profile Phenotypes in African and European Americans: The Jackson Heart Study
Source: PLoS Genet. 2009 Jan 16;5(1):e1000342. doi: 10.1371/journal.pgen.1000342 (PMC2613537; doi:10.1371/journal.pgen.1000342)
Supplement: Table S6 — Multi-SNP models derived using nested regression models and ANOVA for a) TG and b) HDL-C. The most frequent solutions are shown for the 100 runs performed (one run per set of 3300 unrelated individuals). (0.04 MB DOC) [file pgen.1000342.s007.doc]

**Supplementary Table 6: Multi-SNP models derived using nested regression models and ANOVA for a) TG and b) HDL-C.** The most frequent solutions are shown for the 100 runs performed (one run per set of 3300 unrelated individuals).

a) TG

| **SNP Model** | **Relative Frequency** |
| --- | --- |
| rs10096633, rs1031045, rs3779788, rs11995036 | 0.12 |
| rs10096633, rs1031045, rs11995036, rs17482753, rs1011685 | 0.12 |
| rs10096633, rs1031045, rs11995036, rs1569209 | 0.09 |
| rs10096633, rs1031045, rs328 | 0.05 |
| rs10096633, rs1031045, rs328, rs11995036 | 0.05 |

b) HDL-C

| **SNP Model** | **Relative Frequency** |
| --- | --- |
| rs3289, rs13266204, rs13702, rs9644636, rs343, rs2197089, rs258 | 0.12 |
| rs3289, rs13702, rs9644636, rs343, rs1121923, rs2197089, rs258 | 0.08 |
| rs3289, rs13266204, rs13702, rs9644636, rs343, rs2197089 | 0.05 |
| rs3289, rs13702, rs9644636, rs343, rs2197089, rs258 | 0.05 |
| rs13702, rs343, rs3289 | 0.05 |
